# Comorbidity and progression of late onset Alzheimer’s disease: A systematic review

**DOI:** 10.1371/journal.pone.0177044

**Published:** 2017-05-04

**Authors:** Miriam L. Haaksma, Lara R. Vilela, Alessandra Marengoni, Amaia Calderón-Larrañaga, Jeannie-Marie S. Leoutsakos, Marcel G. M. Olde Rikkert, René J. F. Melis

**Affiliations:** 1 Radboud University Medical Center, Radboud Institute for Health Sciences, Department of Geriatric Medicine, Nijmegen, The Netherlands; 2 Radboud University Medical Center, Radboudumc Alzheimer Centre, Nijmegen, The Netherlands; 3 Aging Research Center, Department of Neurobiology, Care Sciences and Society, Karolinska Institutet and Stockholm University, Stockholm, Sweden; 4 Department of Clinical and Experimental Sciences, University of Brescia, Brescia, Italy; 5 EpiChron Research Group on Chronic Diseases, Aragón Health Sciences Institute, IIS Aragón, Miguel Servet University Hospital, Zaragoza, Spain; 6 Red de Investigación en Servicios de Salud en Enfermedades Crónicas, Carlos III Health Institute, Madrid, Spain; 7 Department of Psychiatry, Division of Geriatric Psychiatry and Neuropsychiatry, Johns Hopkins University School of Medicine, Baltimore, Maryland, United States of America; Nathan S Kline Institute, UNITED STATES

## Abstract

**Background:**

Alzheimer’s disease is a neurodegenerative syndrome characterized by multiple dimensions including cognitive decline, decreased daily functioning and psychiatric symptoms. This systematic review aims to investigate the relation between somatic comorbidity burden and progression in late-onset Alzheimer’s disease (LOAD).

**Methods:**

We searched four databases for observational studies that examined cross-sectional or longitudinal associations of cognitive or functional or neuropsychiatric outcomes with comorbidity in individuals with LOAD. From the 7966 articles identified originally, 11 studies were included in this review. The Newcastle-Ottawa quality assessment was used. The large variation in progression measures, comorbidity indexes and study designs hampered the ability to perform a meta-analysis. This review was registered with PROSPERO under DIO: 10.15124/CRD42015027046.

**Results:**

Nine studies indicated that comorbidity burden was associated with deterioration in at least one of the three dimensions of LOAD examined. Seven out of ten studies investigating cognition found comorbidities to be related to decreased cognitive performance. Five out of the seven studies investigating daily functioning showed an association between comorbidity burden and decreased daily functioning. Neuropsychiatric symptoms (NPS) increased with increasing comorbidity burden in two out of three studies investigating NPS. Associations were predominantly found in studies analyzing the association cross-sectionally, in a time-varying manner or across short follow-up (≤2 years). Rarely baseline comorbidity burden appeared to be associated with outcomes in studies analyzing progression over longer follow-up periods (>2 years).

**Conclusion:**

This review provides evidence of an association between somatic comorbidities and multifaceted LOAD progression. Given that time-varying comorbidity burden, but much less so baseline comorbidity burden, was associated with the three dimensions prospectively, this relationship cannot be reduced to a simple cause-effect relation and is more likely to be dynamic. Therefore, both future studies and clinical practice may benefit from regarding comorbidity as a modifiable factor with a possibly fluctuating influence on LOAD.

## 1. Introduction

Dementia is typically defined as a clinical syndrome of cognitive decline that is sufficiently severe to interfere with social or occupational functioning [1]. It is an umbrella term for a group of cognitive disorders characterized by progressive decline in cognitive function interfering with independently carrying out activities of daily life due to brain damage or disease, but not related to delirium or depression [2]. Alzheimer’s disease (AD) is the most common cause of dementia in late life and accounts for 50–70% of the cases [3]. AD is a neurodegenerative syndrome characterized by multidimensional progression consisting of three core dimensions: cognitive, functional and psychiatric symptoms [4], with functional symptoms being defined as a decreased ability to independently perform daily life activities. Its prevalence is increasing rapidly due to aging of most societies, though incidence seems to decline in people with high educational levels in high income countries [5]. This review focuses specifically on late-onset Alzheimer’s disease (LOAD), which is defined as AD with an onset after 65 years of age. LOAD is more prevalent and generally has a more slowly progressing course as compared to early-onset AD [6].

Currently it is impossible to provide patients and their families with a reliable prediction of the course of their disease, as there is substantial variability in rates of decline among individuals with LOAD [7]. Knowing which factors are associated with decline would be useful for understanding and slowing disease progression, as well as for individual prognosis [8]. Potentially influential factors are comorbidities. Comorbidity is defined as any additional co-existing ailment in a patient with a particular index disease [9], in this case LOAD. It has been shown that comorbidities are more prevalent among individuals with LOAD as compared to demographically-matched controls [10]. In addition, a review indicated that comorbidity contributes to decline in LOAD [11]. However, it is unclear exactly how comorbidities affect the separate facets of LOAD, since many studies merely report relations between comorbidity and one dimension of LOAD, despite the multifaceted nature of the syndrome [4]. In order to provide a multidimensional overview, this review investigates whether there is evidence for an association between comorbid disease burden and cognitive, functional and psychiatric symptoms in individuals with LOAD, both cross-sectionally and longitudinally.

## 2. Methods

The articles were identified using the electronic databases Medline, EMBASE, PsycINFO and Cochrane updated until January 2016. Comprehensive search strategies were developed separately for each of the four databases (S1, S2, S3 and S4 Appendixes). The keywords “Alzheimer” and “observational studies” and “progression” and “comorbidity” were used in subsequent combinations with either “cognition” or “daily functioning” or “behavior disorders”, along with their synonyms. In order to meet the inclusion criteria, articles had to examine cognitive or functional or neuropsychiatric outcomes in relation to comorbidity in individuals diagnosed with LOAD (age 65 or over at onset). No restriction for years of publication was used. In order to summarize all available evidence on the association between comorbid disease burden and dementia symptoms, both longitudinal and cross-sectional studies were included. Since this review addresses somatic comorbid disease burden in general, the influence of individual diseases is beyond the scope of this study. The protocol of this review was registered with PROSPERO and can be accessed through DIO: 10.15124/CRD42015027046.

**Fig 1:**
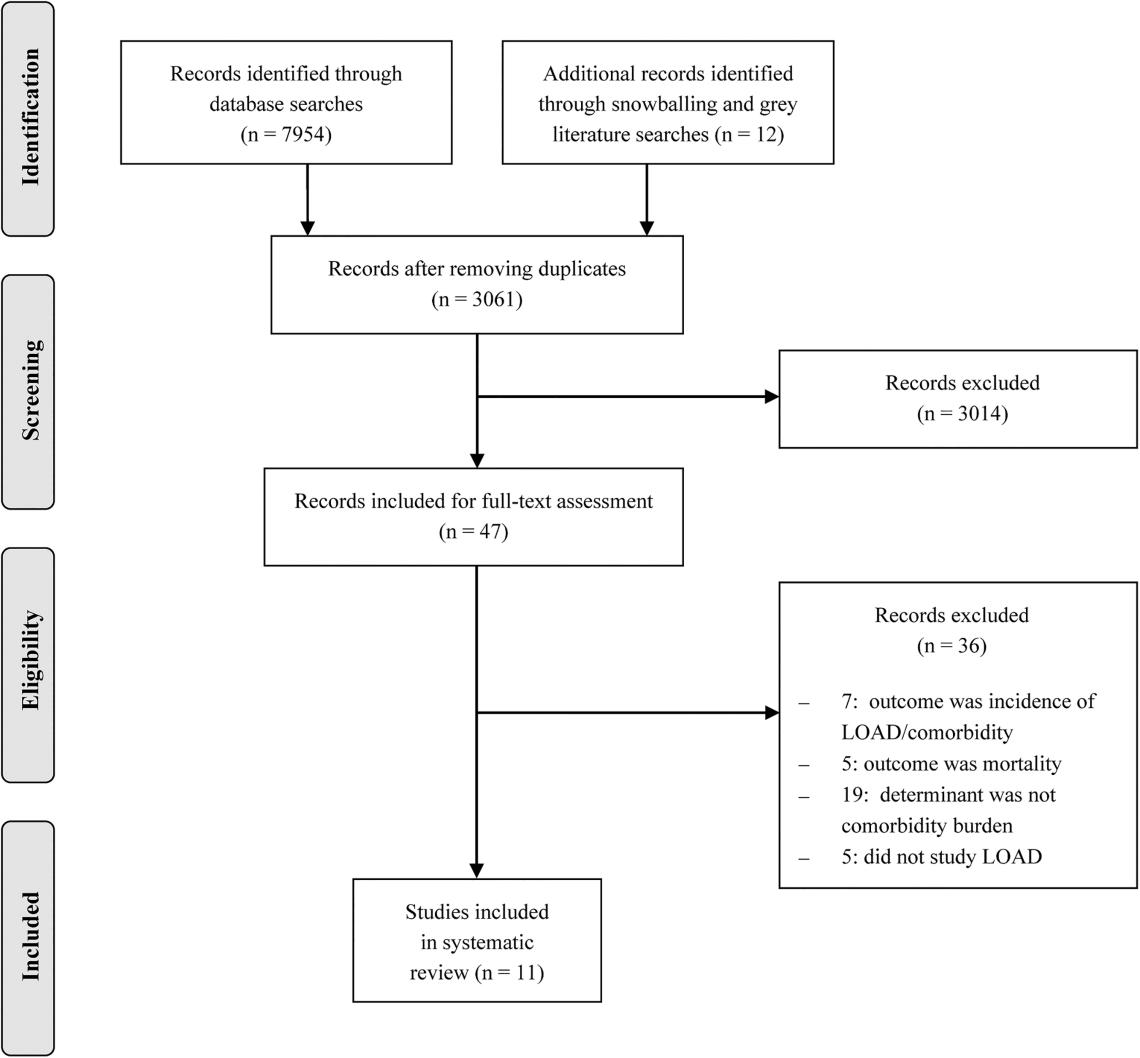
Prisma flow diagram.

The database searches yielded 7954 articles and 12 papers were identified by other means: the snowball method yielded seven articles and an additional search for grey literature in the online databases OpenGrey, Open DOAR and Google Scholar resulted in the identification of five new articles. From the total of 7966 articles originally identified, 4905 duplicates were excluded. The title and abstract of the 3061 articles were independently screened by two reviewers (L.R.V., M.L.H.) and 3014 articles were excluded for not meeting the inclusion criteria. After that, another 36 studies were excluded based on full text assessment which was performed in duplicate as well (L.R.V., M.L.H.). Discrepancies between the two reviewers were resolved by a third reviewer (R.J.F.M.). The remaining 11 articles were critically appraised, again by two independent reviewers (L.R.V., M.L.H.), using the Newcastle-Ottawa quality assessment for cohort studies [12] which assesses the selection of participants, methods to control for confounding and assessment of the outcome. For cross-sectional studies, an adapted version of the scale was used in which questions regarding follow-up were left out. An overview of the selection process is provided in Fig 1. The first two authors of this review (L.R.V. and M.L.H.) did not (co)author any of the included studies. J.S.L. is first author of one of the included studies [13], as is R.J.F.M. [14]. Moreover, A.M., J.S.L., M.G.M.O.R. and R.J.F.M. all co-authored one of the included studies [14–16].

## 3. Results

Out of the 47 studies deemed eligible after screening, 12 did not examine any LOAD dimension as an outcome, 19 did not study the influence of comorbid disease burden and 5 did not focus on LOAD. The remaining 11 articles included in this review were published between 1998 and 2015 [13–23]. An overview of these studies is provided in Table 1. The diagnosis criteria used for LOAD were NINCDS-ADRDA (National Institute of Neurological and Communicative Disorders and Stroke; Alzheimer's Disease and Related Disorders Association) [2] and/or DSM (Diagnostic and Statistical Manual of Mental Disorders) [24]. Comorbid disease burden was measured using the Cumulative Illness Rating Scale for Geriatrics (CIRS-G) [25], Charlson Comorbidity Index (CCI) [26], General Medical Health Rating (GMHR) [15] and some studies counted the comorbidities based on hospital records, physical examination and/or patient/caregiver report. Although the comorbidity measures differed across studies, most studies used a personal approach with either an interview or medical examination by a physician.

**Table 1:** Description of all selected studies.

Author (year)	Participants	Cohort	Study design	Comorbidity measure	Statistical analysis
	Mean age±SD (yr)	Setting	Measurement interval*	Data source	
**Lyketsos et al. (1999)**	N = 344	Clinical cohort,	Cross-sectional	General Medical Health Rating	Pearson’s correlations
	76.5±NA	Baltimore, U.S.A.	NA	Independent assessment by physician	
**Tekin et al. (2001)**	N = 143	Clinical cohort	Cross-sectional	Cumulative Illness Rating Scale for Geriatrics	Pearson’s correlations and stepwise linear regression with baseline covariates
	76.5±7.8	Los Angeles, U.S.A.	NA	NA	
**Doraiswamy et al. (2002)**	N = 679	Mixed cohort**	Cross-sectional	Cumulative Illness Rating Scale for Geriatrics	General Linear Model
	80.7±NA	13 sites in 9 states of the U.S.A.	NA	Medical history and physical examination	
**Formiga et al. (2009)**	N = 289	Clinical cohort	Cross-sectional	Charlson Comorbidity Index	Logistic regression with baseline covariates
	81.0±6.0	Barcelona, Spain	NA	Interview with patient/caregiver and medical records	
**Oosterveld et al. (2014)**	N = 213	Clinical cohort	Cross-sectional	Cumulative Illness Rating Scale for Geriatrics	Pearson’s correlation and linear regression with baseline covariates
	75.0±10.0	3 sites in the Netherlands	NA	Medical examination by physician	
**Aguero-Torres et al. (1998)**	N = 46	Population-based cohort	Longitudinal	Presence of chronic condition (yes/no)	Linear regression for annual rate of change with baseline covariates
	86.0±5.6	Stockholm, Sweden	3 years	Informant interview	
**Boksay et al. (2005)**	N = 40	Clinical cohort	Longitudinal	Comorbidity count	NA
	69.9±3.2	New York, U.S.A.	4 years	NA	
**Solomon et al. (2011)**	N = 102	Clinical cohort	Longitudinal	Cumulative Illness Rating Scale for Geriatrics	Ordinal logistic regression for categories of decline based on annual rate of change with baseline covariates
	75.4±8.2	Bucharest, Romania	2 years	Medical records and reports of patients/caregivers	
**Leoutsakos et al. (2012)**	N = 230	Population-based cohort	Longitudinal	General Medical Health Rating	Quadratic mixed models with random effects for intercept and time including both baseline and time-varying covariates
	85.9±6.3	Utah, U.S.A.	6 months (time-varying) 11 years (time-invariant)	Interview with patient and caregiver and medical records	
**Melis et al. (2013)**	N = 251	Population-based cohort	Longitudinal	Presence of 0, 1 or ≥2 chronic conditions	Quadratic mixed models with baseline covariates and random effects for intercept and time
	85.5±4.5	Stockholm, Sweden	3–12 years	Stockholm Inpatient Register	
**Aubert et al. (2015)**	N = 170	Clinical cohort	Longitudinal	Charlson Comorbidity Index	Logistic regression with baseline covariates
	83.3±5.4	Nantes, France	1 year	NA	

* = Time between comorbidity measurement and analyzed progression.

** = clinical, retirement and nursing home population.

N = number of participants included in the analysis.

NA = information not available.

Five studies were cross-sectional and six were longitudinal studies with maximum follow-up ranging from 1 to 12 years. The overall mean age of the participants across studies was 80.3 years (range: 69.9–86.0). After quality assessment, only one study was deemed to be of low quality with a score below 50% due to lack of correction for confounding and unrepresentative sampling [19]. Adjustment for baseline age was unclear in two studies [15, 19]. Adjustment for education was lacking or unclear in five studies examining cognition [16, 17, 19–21]. Adjustment for baseline symptoms of interest was unclear in one out of six longitudinal studies [19]. An overview of the quality assessment of all included studies is provided in S5 Appendix.

Nine studies indicated that symptoms were worse in at least one of the three dimensions of LOAD in individuals with increased comorbid disease burden, while two studies failed to find an association. The following sections will address the results for three different dimensions of LOAD.

### 3.1 Studies on global and cognitive abilities

Four cross-sectional and six longitudinal studies examined cognitive outcomes. An overview of these studies, including their measurement scales and results is provided in Table 2. Eight studies used the Mini-Mental State Examination (MMSE) [27] to measure cognition, one used the Global Deterioration Scale (GDS) [28] and one used a composite score of neuropsychological tests [16]. The presence of comorbidities was related to decreased cognitive abilities in seven out of the ten studies examining cognition. Three of these were cross-sectional and four were longitudinal studies. In one of the longitudinal studies only time-varying comorbidity burden, not time-invariant baseline comorbidity, was associated with decreased cognitive abilities (Table 2).

**Table 2:** Description of studies on global and cognitive abilities.

Author (year)	Cognitive measure	Study design	Estimate (SE) **	Results
		Measurement interval*		
**Tekin et al.** **(2001)**	Mini Mental State Examination	Cross-sectional	NA	Cumulative Illness Rating Scale for Geriatrics was not related to cognitive status
		NA		
**Doraiswamy et al. (2002)**	Mini Mental State Examination	Cross-sectional	R^2^ = 0.34, *p<0*.*0001*	Higher Cumulative Illness Rating Scale for Geriatrics was related to lower cognitive status
		NA		
**Formiga et al.** **(2009)**	Mini Mental State Examination	Cross-sectional	Mean Δ = 0.2 (0.11), *p = 0*.*02*	Higher Charlson Comorbidity Index was related to lower cognitive status
		NA		
**Oosterveld et al. (2014)**	Neuropsychological assessment	Cross-sectional	r = −0.19, *p<0*.*01*	Higher Cumulative Illness Rating Scale for Geriatrics was related to poorer cognitive status
		NA		
**Aguero-Torres et al. (1998)**	Mini Mental State Examination	Longitudinal	b = -0.25 (0.64), *p = 0*.*694*	The presence of a chronic condition was not related to changes in cognition
		3 years		
**Boksay et al.** **(2005)**	Global Deterioration Scale	Longitudinal	Mean Δ = 2.1 (1.04), *p<0*.*05*	The presence of a higher number of comorbidities was related to a faster decline in cognition
		4 years		
**Solomon et al. (2011)**	Mini Mental State Examination	Longitudinal	b = 0.01, *p = 0*.*02*	Higher Cumulative Illness Rating Scale for Geriatrics was related to faster decline in cognition
		2 years		
**Leoutsakos et al. (2012)**	Mini Mental State Examination	Longitudinal	β = −1.07 (0.42),*p = 0*.*01*	Lower General Medical Health Rating was related to a decreased cognitive abilities when analyzing it as a time-varying covariate, but not when using it as a time invariant baseline covariate
		6 months (time-varying)		
		11 years (time-invariant)	β = 0.23 (0.46), *p = 0*.*61*	
**Melis et al.** **(2013)**	Mini Mental State Examination	Longitudinal	b = -0.27, *p = 0*.*17*	The presence of either 0, 1 or ≥2 comorbidities was not related to cognition cross-sectionally nor longitudinally.
		3–12 years		
**Aubert et al.** **(2015)**	Mini Mental State Examination	Longitudinal	OR = 1.30, CI: 1.02–1.65, *p = 0*.*03*	Higher Charlson Comorbidity Index was related to faster decline in cognition
		1 year		

NA = Not available. SE = Standard Error. CI = 95% Confidence Interval.

* Time between comorbidity measurement and analyzed progression.

** For mixed models the interaction with the linear slope is reported.

### 3.2 Studies on daily functioning

Four cross-sectional and three longitudinal studies examined daily functioning. These studies are summarized in Table 3. Functional impairment was measured using (instrumental) Activities of Daily Living ((i)ADL) [29] in four studies, the Clinical Dementia Rating Scale (CDR) [30] in one study, Health Utilities Index subscales [31] in one study and the Psychogeriatric Dependency Rating Scale (PGDRS) [32] in one last study. Five out of the seven studies examining daily functioning found comorbidities to be related to lower functional abilities. Three of these were cross-sectional and two were longitudinal. In one of the longitudinal studies only time-varying comorbidity burden, not time-invariant baseline comorbidity burden, was associated with functional decline [13]. In the longitudinal study conducted by Melis et al. (2013) also a cross-sectional association between baseline comorbidity burden and baseline functional status was found [33]. The two smallest studies (one longitudinal, one cross-sectional) did not find an association [17, 23].

**Table 3:** Description of studies on daily functioning.

Author (year)	Daily functioning measure	Study design	Estimate (SE) **	Results
		Measurement interval*		
**Lyketsos et al. (1999)**	Psychogeriatric Dependency Rating Scale	Cross-sectional	β = -4.1 (0.68), *p = 0*.*001*	Lower General Medical Health Rating was related to increased functional impairment
		NA		
**Tekin et al.** **(2001)**	(instrumental) Activities of Daily Living	Cross-sectional	r = 0.10, *p = 0*.*991*	Cumulative Illness Rating Scale for Geriatrics was not related to functioning
		NA		
**Doraiswamy et al. (2002)**	Subscale for self-care of the Health Utilities Index	Cross-sectional	R^2^ = 0.47, *p<0*.*0001*	Higher Cumulative Illness Rating Scale for Geriatrics was related to greater functional impairment
		NA		
**Oosterveld et al. (2014)**	(instrumental) Activities of Daily Living	Cross-sectional	r = −0.37, *p<0*.*001*	Higher Cumulative Illness Rating Scale for Geriatrics was related to lower functional status
		NA		
**Solomon et al. (2011)**	(instrumental) Activities of Daily Living	Longitudinal	OR = 2.7, CI: 0.7–9.6	Higher Cumulative Illness Rating Scale for Geriatrics tended to be related to increased decline in functioning, but this was not significant
		2 years		
**Leoutsakos et al. (2012)**	Clinical Dementia Rating sum of boxes	Longitudinal	β = 1.79 (0.34), *p<0*.*001*	Lower General Medical Health Rating was related to decreased functioning when analyzing it as a time-varying covariate, but not when using it as a time invariant baseline covariate
		6 months (time-varying)		
		11 years (time-invariant)	β = −0.51 (0.34), *p = 0*.*13*	
**Melis et al.** **(2013)**	Activities of Daily Living	Longitudinal	b = 0.34, *p = 0*.*006*	Presence of ≥2 chronic conditions at baseline was cross-sectionally related to lower baseline functional status and also longitudinally to faster decline in functioning
		3–12 years		

NA = Not available. SE = Standard Error. CI = 95% Confidence Interval.

* Time between comorbidity measurement and analyzed progression.

** For mixed models the interaction with the linear slope is reported.

### 3.3 Studies on neuropsychiatric symptoms

As shown in Table 4, all three studies examining neuropsychiatric symptoms (NPS) used the Neuropsychiatric Inventory (NPI) [34]. Two studies were cross-sectional and one was longitudinal. Two studies (one cross-sectional, one longitudinal) found comorbidities to be related to increased NPS. In the one longitudinal study, only time-varying comorbidity burden, not time-invariant baseline comorbidity burden, was associated with more NPS. One cross-sectional study found no association [17].

**Table 4:** Description of studies on neuropsychiatric symptoms.

Author (year)	Neuropsychiatric measure	Study design	Estimate (SE) **	Results
		Measurement interval*		
Tekin et al. (2001)	Neuropsychiatric Inventory	Cross-sectional	R = 0.21, *p<0*.*05*	Cumulative Illness Rating Scale for Geriatrics was not related to neuropsychiatric symptoms
		NA		
Oosterveld et al. (2014)	Neuropsychiatric Inventory	Cross-sectional	R = 0.20, *p<0*.*001*	Higher Cumulative Illness Rating Scale for Geriatrics was related to more neuropsychiatric symptoms
		NA		
Leoutsakos et al. (2012)	Neuropsychiatric Inventory	Longitudinal	4.57 (1.80), *p = 0*.*01*	Lower General Medical Health Rating was related to more neuropsychiatric symptoms when analyzing it as a time-varying covariate, but not when using it as a time invariant baseline covariate
		6 months (time-varying)		
		11 years (time-invariant)	0.93 (1.05), *p = 0*.*38*	

*NA = Not available*. SE = Standard Error.

* Time between comorbidity measurement and analyzed progression.

** For mixed models the interaction with the linear slope is reported.

## 4. Discussion

### 4.1 Discussion of the current scientific evidence

Although the total evidence available for this review was limited, the main finding was that increased somatic comorbid disease burden was associated with increased cognitive, functional and neuropsychiatric symptoms in LOAD. Nine studies showed an association between comorbid disease burden and LOAD symptoms, while only two studies found no association. Primarily cross-sectional associations were found between comorbidity burden and the three dimensions of LOAD. In the prospective studies, evidence for longitudinal associations between baseline comorbidity burden and LOAD progression was inconsistent. Only three studies examined all dimensions of LOAD simultaneously and two of these found associations between comorbidity burden and all dimensions of LOAD [13, 16]. The third study by Tekin et al. was cross-sectional and did not find any associations at all [17]. It was argued that the associations were possibly obscured by the relatively low levels of comorbidity (mean±SD CIRS-G: 5.8±3.0) and the young age (mean±SD: 76.5±7.8 years) of the study population. Only one study evaluated time-varying associations and found all three dimensions to be associated with time-varying comorbidity burden.

Seven out of ten studies examining cognitive abilities found a significant association between increased comorbid disease burden and lower cognitive abilities. Of the three studies which found no association, two were longitudinal and conducted in the same cohort [14, 22]. The third was the study by Tekin et al. (2001) mentioned previously [17]. Five out of seven studies examining daily functioning found decreased functional status to be associated with comorbid disease burden. The two negative studies were the study by Tekin et al. (2001) and the smallest study on daily functioning by Solomon et al., which detected a non-significant trend [17, 23]. Two out of three studies examining NPS found comorbidities to be related to increased NPS. The negative study once again being the study by Tekin et al. (2001) [17].

The overall quality of the available evidence was rated as high according to the Newcastle-Ottawa quality assessment (S5 Appendix), with the exception of one study of which the results should be interpreted with caution [19]. It should be noted that this assessment does not distinguish between longitudinal and cross-sectional studies and that 5 out of the 11 studies in this review were cross-sectional. The (reporting of) adjustment for confounders was found to be inconsistent across studies and although most studies adjusted for age, the correction for education was far less common. It is worth mentioning that the quality assessment does not assess the studies’ sample size (Table 1) and although it takes follow-up rate at the end of the study into account, the length of the study is ignored, while these two things are clearly related.

Altogether, the majority of the studies in this review suggest that comorbidity contributes to a (more rapid) worsening of symptoms in LOAD, which is in accordance with a broader conceptualization of LOAD as the consequence of a dynamic interaction of disease-related and individual factors [35]. However, the association between comorbidity burden and LOAD progression cannot be reduced to a linear causal relation where exposures to higher levels of comorbidity burden are linked prospectively to dementia outcomes. Rather their relation is more complex and dynamic. We will expand on this in the next paragraph.

### 4.2 Observing associations over time

The study by Leoutsakos et al. (2012) was the only study that used both time-invariant and time-varying covariates, while the other studies analyzed comorbid disease burden as a baseline, time-invariant predictor only. Analyzing comorbid disease burden as a time-varying covariate means looking at the relationship between comorbidity and LOAD facets at corresponding points over time. Comparing these two analysis approaches offered an interesting perspective; when analyzing the association between baseline medical health rating and subsequent LOAD progression no significant relationship was found, however, upon examination of the rating as a time-varying covariate, a clear association with cognitive, functional and psychiatric progression emerged [13]. Based on these results Leoutsakos et al. (2012) postulated that the association between comorbid disease burden and LOAD progression is an immediate, proximal one; how patients are doing on the facets of LOAD at a given point in time is affected by their health at that time.

The results of this review provide support for this notion, since associations were predominantly found in studies analyzing cross-sectional associations or near future progression and were rarely observed in studies analyzing progression in the distant future. For example, when examining the studies on comorbidity and cognition, six out of seven studies reporting an association measured cognition cross-sectionally or in the near future. In other words, the time between comorbidity measurement and analyzed symptoms (from now on referred to as ‘measurement interval’) in these studies was shorter than 2 years (Table 1). For the extent to which longitudinal studies with measurement intervals longer than 2 years were available [13, 14, 19, 22], the evidence for associations between baseline comorbidity burden and cognitive progression was lacking. A possible explanation for this pattern could be that comorbidity status fluctuates, which could dilute the relationship between baseline comorbid disease burden and cognitive progression over time.

In short, the association of comorbidity and cognition appears to be time-varying, i.e. dynamic. This hypothesis is supported by previous studies with a more specific scope of vascular co-morbidities, which found associations between change in comorbidity measures and progression of cognitive impairment [36–38]. Using comorbidity as a time-varying covariate could possibly elucidate the relationship with progression by taking fluctuations in comorbidity status across follow-up into account. Although it is generally accepted that proving causality is hardly possible in observational studies, it must be noted that this is particularly so in time-varying analysis, since the establishment of a temporal relationship is impossible. Another important methodological consequence of studying occurrence relations in a time-varying way is that levels of exposure to possible risk factors need to be established at all follow-ups. This predictor information may not be available at follow-up assessments or be assessed with less rigor or detail in cohort studies.

### 4.3 Potential mechanisms of the effect of comorbidity

The potential mechanisms through which comorbidity may affect the different dimensions of LOAD are numerous. For example, comorbidity is often associated with increased stress levels and it has been shown that stressful experiences are associated with increased risk of dementia, possibly through an imbalance in the adrenocortical axis [3]. A study in mice showed that short stress simulating conditions may exacerbate cognitive deficits in preclinical LOAD by accelerating amyloid pathology and reducing synapse numbers [39]. Another common feature of comorbidity is disrupted sleep, which is known to increase the risk of cognitive impairment [40]. Especially in people with LOAD, sleep deprivation may contribute to worse cognitive function and more NPS, since LOAD itself is already associated with a delay in circadian phase [41, 42]. In line with our findings, a detrimental influence of comorbidities on cognition was also observed in people with Parkinson’s disease [43]. A prospective study including 294 elderly showed that excessive polypharmacy, a logical consequence of increased comorbid disease burden, is also associated with decreased cognitive capacity [7]. Moreover, polypharmacy has been found to contribute to functional decline in community-dwelling patients with LOAD [44]. The fact that comorbid disease burden appears to be positively associated with both cognitive and functional decline is not surprising given the two are strongly related in LOAD [1]. In fact, functional impairment in AD appears to follow cognitive impairment temporally [45, 46]. Impaired daily functioning, in turn, may decrease the amount of leisure and social activities, which are known to be protective against cognitive decline [47], or lower levels of other healthy behaviors and self-care. Similar mechanisms as stated in the previous paragraph could therefore drive the patient into a negative spiral of both functional and cognitive deterioration. A relationship between disease burden and daily functioning was also observed in several large population-based studies in healthy elderly [48]. This indicates that comorbid conditions may lead to functional impairment irrespective of LOAD diagnosis, for example through conditions affecting mobility. Although the number of studies supporting an association between comorbidities and increased NPS in LOAD is small, multiple studies in different populations support the relation between increased comorbid disease burden and psychiatric disorders such as anxiety and depression [8, 49]. The cognitive and functional consequences of increased comorbid disease burden are likely to contribute to social deprivation and stress, which were found to be significant predictors of psychosis and depression [50].

### 4.4 Comparability of included studies

During the critical appraisal of the studies, some methodological challenges emerged. The divergent sampling frame was one of them. Three of the studies were population-based, while seven used a clinical sample and one study sample was obtained in multiple settings (Table 1). Although heterogeneity can increase external validity, differences in study setting can also potentially influence the results due to differences in case-mix. However, comparing the population based and non-population-based studies which analyzed similar dimensions, no pattern of discrepancy was found in the results. Thus, the association between somatic comorbidities and progression appears to be independent of study setting.

Differences in inclusion criteria among studies could also affect the case-mix and therefore hinder comparability between studies. Only the study by Boksay et al. (2005) used stringent criteria by excluding patients with history of various comorbidities, which might hamper comparability, along with its small sample size and lack of correction for age, education and baseline cognitive status in its analyses.

Four studies did not analyze results for LOAD separately from other dementia types [14, 15, 20, 22]. Since all four studies reported the majority of their sample to be suffering from LOAD and their results appeared to be in accordance with the other studies included, this probably had only minor influence on our results.

Given the high quality of the cross-sectional studies, we decided to include both longitudinal and cross-sectional studies in this review to provide an integrated overview of all available studies on this topic. Although the cross-sectional studies do not address disease progression over time, they were mostly in line with the longitudinal studies using measurement intervals ≤2 years.

### 4.5 Strengths and limitations

One of the strengths of this review is its multidimensional approach. As it is unambiguous that patients also experience impairment in their activities of daily living and behavioral changes, LOAD cannot be defined as cognitive impairment alone [4]. The narrow approach of LOAD progression adopted in many studies is therefore worrisome. By taking into account all three dimensions this review provides a more comprehensive perspective on LOAD.

A limitation of this review is the large variation in progression measures, comorbidity indexes, data sources and study designs of the included studies (Table 1), which hampers quantification of the association between comorbid disease burden and progression. The differences between study populations also pose a problem, given the large heterogeneity in age, gender, stage of LOAD and medication use. Moreover, it is questionable whether all measures of a certain dimension truly reflect the same construct or whether the comorbidity measures were sensitive enough to capture comorbidity burden with sufficient detail. Therefore, no meta-analysis could be performed.

Another limitation of this review is that none of its studies took the onset or duration of comorbidities into account. It might be not so much the amount or severity of the comorbidity burden that is relevant, but rather the length of the exposure to comorbidity burden. Without this information, this hypothesis is impossible to be verified or refuted. Given that dementia has a long period of insidious onset, reverse causation cannot be ruled out, not even in the studies which focused on the effect of comorbidity burden at the point where LOAD became incident.

In contrast to many other studies which focus on a single comorbid condition, this review aimed to examine the relation of overall somatic comorbid disease burden with LOAD progression, providing a more generalizable overview. However, a drawback of using scales for overall comorbid disease burden is the potential circularity in the studies examining NPS, caused by the presence of a psychiatric domain in some of the comorbidity measures, such as CIRS-G. The choice to study overall comorbidity burden also renders it impossible to draw conclusions about individual comorbidities. Understanding the mechanisms of and the extent to which singular comorbidities affect the course of LOAD might enable us to slow down multidimensional progression and provide a more individualized prognosis. However, this was beyond the scope of this review.

### 4.6 Conclusion

It is evident that cognitive, functional and psychiatric decline should be addressed simultaneously to obtain a more comprehensive understanding of LOAD progression. One of our first observations is that, although highly relevant, only three studies have addressed these three dimensions of LOAD simultaneously. Therefore, additional multidimensional longitudinal studies are needed to provide a scientific basis for evidence-based care of the growing number of individuals affected with LOAD and comorbid diseases.

This review provides evidence of an association between somatic comorbidities and multifaceted LOAD progression. Given that comorbid disease burden at a given time point seems to be associated with LOAD facets around that same moment, while not in the long term, it seems likely that there is a dynamic relationship between comorbid disease burden and LOAD progression.

With comorbidity burden being a possibly modifiable factor contributing to LOAD, these results stress the importance of optimal treatment and monitoring of comorbidities in people with LOAD. Monitoring progression might be particularly important in case somatic comorbidities manifest after LOAD onset, given the association between comorbidities and LOAD progression in the near future. Prevention and accurate treatment of comorbidities by health care professionals may prevent rapid progression of LOAD.

Moreover, this review emphasizes the relevance of taking comorbidity burden into account when investigating LOAD progression, not only at diagnosis, but also at follow-up. Future studies might gain more knowledge by considering the possibility of a dynamic interconnection between comorbid disease burden and LOAD progression, by means of repeatedly measuring comorbidity status from the preclinical phase of LOAD onwards and analyzing it as a time-varying covariate.

Concluding, there is a clear association between comorbid disease burden and LOAD progression and this association cannot be reduced to a simple, linear cause-effect relation. Therefore, both future studies and clinical practice may benefit from regarding comorbidity as a modifiable factor with a possibly fluctuating influence on LOAD.

## Supporting information

S1 Appendix Search strategy Medline. (PDF)

S2 Appendix Search strategy PsycINFO. (PDF)

S3 Appendix Search strategy EMBASE. (PDF)

S4 Appendix Search strategy Cochrane. (PDF)

S5 Appendix Quality assessment. (PDF)

S6 Appendix PRISMA checklist. (PDF)

S7 Appendix Review protocol. (PDF)
