# Supplementary material for: Comorbidity and progression of late onset Alzheimer’s disease: A systematic review
Source: PLoS One. 2017 May 4;12(5):e0177044. doi: 10.1371/journal.pone.0177044 (PMC5417646; doi:10.1371/journal.pone.0177044)
Supplement: S1 Appendix — (PDF) [file pone.0177044.s001.pdf]

S1 Appendix. Search strategy Medline.

Medline tools:
- kf: keyword heading word
- kw: keyword heading
- ti: title
- ab: abstract
- tw (ti+ab): text word

1) Alzheimer's Disease

1. exp Alzheimer Disease/ or alzheimer*.sh.
2. (alzheimer sclerosis or alzheimer disease late onset or alzheimer type dementia or dementia primary senile degenerative or presenile alzheimer dementia or alzheimer syndrome or dementia alzheimer-type or senile dementia or dementia alzheimer or dementia alzheimer type or presenile dementia or dementia senile or late onset alzheimer disease or alzheimer's disease or primary senile degenerative dementia or syndrome alzheimer or dementia presenile or alzheimer disease assessment scale or alzheimer's disease or alzheimer's-disease or alzheimer-disease).tw.
3. (alzheimer sclerosis or alzheimer disease late onset or alzheimer type dementia or dementia primary senile degenerative or presenile alzheimer dementia or alzheimer syndrome or dementia alzheimer-type or senile dementia or dementia alzheimer or dementia alzheimer type or presenile dementia or dementia senile or late onset alzheimer disease or alzheimer's disease or primary senile degenerative dementia or syndrome alzheimer or dementia presenile or alzheimer disease assessment scale or alzheimer's disease or alzheimer's-disease or alzheimer-disease).kf.
4. exp Dementia/ or dementia.sh.
5. (amentia* or dement* senile paranoid or dementia presenile or mental deterioration).tw.
6. (amentia* or dement* senile paranoid or dementia presenile or mental deterioration).kf.
7. 1 or 2 or 3 or 4 or 5 or 6

2) Observational/Prognosis/Predictor/Comorbidity

8. "Observational studies".ti. or epidemiologic studies/ or exp case-control studies/ or cross-sectional studies/
9. ((case adj3 control) or (cohort adj5 (study or studies or analy$)) or (follow-up adj5 (study or studies)) or (longitudinal or retrospective or prospective or (cross adj5 sectional)) or (observational adj5 (study or studies))).af.
10. 8 or 9
11. 7 and 10
12. exp Prognosis/ or prognos*.sh.
13. exp Probability/ or probab*.sh.
14. exp Proportional Hazards Models/
15. exp Likelihood Functions/
16. exp Logistic Models/
17. exp Forecasting/ or forecast*.sh.
18. exp Risk Factors/ or risk factor*.sh.
19. (decision support techniques or prediction or predictive validity or predictor variable).tw.
20. (decision support techniques or prediction or predictive validity or predictor variable).kf.
21. ((risk adj prediction) or (predictor adj variabl??) or (increas* adj risk)).mp. or ((risk adj assessement?) or (predict* adj risk?) or (risk adj factor?) or (validat* or predict* or rule*)).tw.
22. ((risk adj prediction) or (predictor adj variabl??) or (increas* adj risk)).mp. or ((risk adj assessement?) or (predict* adj risk?) or (risk adj factor?) or (validat* or predict* or rule*)).kf.
23. ((predict* and (outcome* or risk* or model*)) or ((history or variable* or criteria or scor* or characteristic* or finding* or factor*) and (predict* or model* or decision* or identi* or prognos*)) or (decision* and (model* or clinical* or (logistic adj3 models)))).tw. or ((predict* and (outcome* or risk* or model*)) or ((history or variable* or criteria or scor* or characteristic* or finding* or factor*) and (predict* or model* or decision* or identi* or prognos*)) or (decision* and (model* or clinical* or (logistic adj3 models)))).kf.
24. 12 or 13 or 14 or 15 or 16 or 17 or 18 or 19 or 20 or 21 or 22 or 23
25. exp Comorbidity/ or comorbidit*.sh.
26. (multimorbidit* or comorbidit*).tw.
27. (multimorbidit* or comorbidit*).kf.
28. (co morbidit* or co-morbidit* or coomorbidit*).tw.
29. (co morbidit* or co-morbidit* or coomorbidit*).kf.
30. (chronic diseases or cooccurring diseases or co-occurring diseases or co occurring diseases or clusters of diseases or disease burden or physical health or medical health or charlson or cumulative illness scale geriatrics or polymorbidity or disease count).tw.
31. (chronic diseases or cooccurring diseases or co-occurring diseases or co occurring diseases or clusters of diseases or disease burden or physical health or medical health or charlson or cumulative illness scale geriatrics or polymorbidity or disease count).kf.
32. 25 or 26 or 27 or 28 or 29 or 30 or 31
33. 24 and 32

3) Multidimensional progression

34. exp Cognition/ or cognit*.sh.
35. exp Cognition Disorders/ or cognit* disorder*.sh.
36. cognit*.tw.
37. cognit*.kf.
38. (consciousness disorders or overinclusion or cognitive performance or confusion or intellectual disability or perceptual disorders or mental competency or perception or thinking or aptitude or mild cognitive impairment or cognitive defect or cognitive generalization or cognitive complexity or cognitive contiguity or cognitive dissonance or cognitive appraisal or cognitive maps or wayfinding or spatial imagery or direction perception or thought content or cognitive functioning or executive functioning or intellectual functioning or mathematical ability or reading ability or verbal ability or cognitive deficits or cognitive dysfunction or executive dysfunction or thought disturbances or human information process or cognitive science or information processing model or metacognition or intelligence or intelligence measures or intelligence quotient).tw. or (consciousness disorders or overinclusion or cognitive performance or confusion or intellectual disability or perceptual disorders or mental competency or perception or thinking or aptitude or mild cognitive impairment or cognitive defect or cognitive generalization or cognitive complexity or cognitive contiguity or cognitive dissonance or cognitive appraisal or cognitive maps or wayfinding or spatial imagery or direction perception or thought content or cognitive functioning or executive functioning or intellectual functioning or mathematical ability or reading ability or verbal ability or cognitive deficits or cognitive dysfunction or executive dysfunction or thought disturbances or human information process or cognitive science or information processing model or metacognition or intelligence or intelligence measures or intelligence quotient).kf.
39. exp Spatial Memory/ or exp Memory/ or exp Memory, Long-Term/ or exp Memory, Episodic/ or exp Memory Disorders/ or exp Memory, Short-Term/ or (spatial memory or memory or memory, long-term or memory, episodic or memory disorder* or memory, short-term).sh.
40. (spatial memory disorders or memory disorder or memory losses or memory disorders age related or retention disorder cognitive or memory disorder semantic or age-related memory disorder or memory deficits or spatial memory disorder or cognitive retention disorder or memories autobiographical or memories episodic or memories prospective or longterm memories or memories remote or memory long-term or immediate memories or short-term memory or working memory or memory shortterm or recall immediate or verbal memory or visual memory or amnesia or anterograde amnesia or global amnesia or retrograde amnesia or memory consolidation or memory decay or memory trace or visuospatial memory or associative memory or auditory memory or false memory or olfactory memory or recognition or reference memory or repetition priming or retrospective memory or sensory memory or tactile memory or memory bias or word list recall or word recognition or working memory).tw. or (spatial memory disorders or memory disorder or memory losses or memory disorders age related or retention disorder cognitive or memory disorder semantic or age-related memory disorder or memory deficits or spatial memory disorder or cognitive retention disorder or memories autobiographical or memories episodic or memories prospective or longterm memories or memories remote or memory long-term or immediate memories or short-term memory or working memory or memory shortterm or recall immediate or verbal memory or visual memory or amnesia or anterograde amnesia or global amnesia or retrograde amnesia or memory consolidation or memory decay or memory trace or visuospatial memory or associative memory or auditory memory or false memory or olfactory memory or recognition or reference memory or repetition priming or retrospective memory or sensory memory or tactile memory or memory bias or word list recall or word recognition or working memory).kf.
41. exp Language/ or exp Language Disorders/ or exp Speech-Language Pathology/ or (language or language disorders or speech-language pathology).sh.
42. exp Verbal Behavior/ or verbal behavior.sh.
43. (language disorder acquired or pathology speech or pathology language or verbal fluency or "speech and language" or language or linguistic or "speech and language assessment" or language ability or oral communication or speech analysis or speech perception or speech disorder or language disability or language ability or language test or communication disorders).tw. or (language disorder acquired or pathology speech or pathology language or verbal fluency or "speech and language" or language or linguistic or "speech and language assessment" or language ability or oral communication or speech analysis or speech perception or speech disorder or language disability or language ability or language test or communication disorders).kf.
44. exp Executive Function/ or executive function*.sh.
45. exp Attention/ or attention.sh.
46. exp Problem Solving/ or problem solving.sh.
47. exp Decision Making/ or decision making.sh.
48. exp Orientation/ or orientation.sh.
49. exp Reading/ or reading.sh.
50. exp Judgment/ or judgment.sh.
51. (executive control or concentration or shared decision making or mental speed or verbal reasoning or abstraction).tw. or (executive control or concentration or shared decision making or mental speed or verbal reasoning or abstraction).kf.
52. 34 or 35 or 36 or 37 or 38 or 39 or 40 or 41 or 42 or 43 or 44 or 45 or 46 or 47 or 48 or 49 or 50 or 51

53. exp Motor Activity/ or exp "Activities of Daily Living"/ or activit* of daily living.sh. or motor activit*.sh.
54. exp Self Care/ or self care.sh.
55. exp Walking/ or walking.sh.
56. exp Locomotion/ or locomot*.sh.
57. exp Postural Balance/ or postural balance*.sh.
58. exp Hand Strength/ or hand strength.sh.
59. exp Physical Endurance/ or physical endurance.sh.

60. exp Motor Activity/
61. exp Motor Skills/ or motor skill*.sh.
62. exp Gait/ or exp Gait Disorders, Neurologic/ or (gait or gait disorder*).sh.
63. exp Compressive Strength/ or exp Muscle Strength/ or (compressive strength or muscle strength).sh.
64. exp Accidental Falls/ or accidental fall*.sh.
65. exp Personal Autonomy/ or personal autonomy.sh.
66. exp Work Capacity Evaluation/ or work capacity evaluation.sh.
67. (physical function or ambulation or limitation of activity chronic or self management or self-care or self-management or musculoskeletal equilibrium or postural equilibrium or grasps or grips or pinch strength or (physical function or ambulation or limitation of activity chronic or self management or self-care or self-management or musculoskeletal equilibrium or postural equilibrium or grasps or grips or pinch strength) or (physical function or ambulation or limitation of activity chronic or self management or self-care or self-management or musculoskeletal equilibrium or postural equilibrium or grasps or grips or pinch strength)).tw. or (physical function or ambulation or limitation of activity chronic or self management or self-care or self-management or musculoskeletal equilibrium or postural equilibrium or grasps or grips or pinch strength).kf.
68. 53 or 54 or 55 or 56 or 57 or 58 or 59 or 60 or 61 or 62 or 63 or 64 or 65 or 66 or 67

69. exp Mental Disorders/ or mental disorder*.sh.
70. exp Geriatric Psychiatry/ or *Psychiatry/ or geriatr* psychiat*.sh. or psychiatr*.sh.
71. exp Behavioral Symptoms/ or behavior symptom*.sh.
72. exp Anxiety/ or exp Anxiety Disorders/ or (anxiety or anxiety disorder*).sh.
73. confusion/ or exp delirium/ or (confusion or delirium).sh.
74. emotions/ or affect/ or anger/ or apathy/ or hostility/ or (emotions or affect or anger or apathy or hostility).sh.
75. exp Delirium, Dementia, Amnestic, Cognitive Disorders/ or exp Psychotic Disorders/ or (delirium, dementia, amnestic, cognitive disorders or psychotic disorders).sh.
76. exp Aggression/ or aggress*.sh.
77. exp Hostility/
78. exp Behavior Control/ or exp Illness Behavior/ or exp Social Behavior Disorders/ or (behavior control or illness behavior or social behavior disorders).sh.
79. exp Delusions/ or delusion*.sh.
80. exp Hallucinations/ or hallucination*.sh.
81. exp Psychomotor Agitation/ or psychomotor agitation.sh.
82. exp Depression/ or depression.sh.
83. exp Euphoria/ or euphoria.sh.
84. exp Apathy/ or apathy.sh.
85. exp Irritable Mood/ or irritable mood.sh.
86. (emotional disorder or mental patient or mental instability or mood disorder or organic psychosyndrome or dysphoria or neuropsychological assessment or psychiatric evaluation or psychiatric disorders or indifference or disinhibition or lability or aberrant motor behavior or hallucinat* or agitat* or anxiety elation or apath* or neuropsychiat*).tw. or (emotional disorder or mental patient or mental instability or mood disorder or organic psychosyndrome or dysphoria or neuropsychological assessment or psychiatric evaluation or psychiatric disorders or indifference or disinhibition or lability or aberrant motor behavior or hallucinat* or agitat* or anxiety elation or apath* or neuropsychiat*).kf.
87. 69 or 70 or 71 or 72 or 73 or 74 or 75 or 76 or 77 or 78 or 79 or 80 or 81 or 82 or 83 or 84 or 85 or 86

88. exp Disease Progression/ or disease progress*.sh.
89. exp Patient Outcome Assessment/ or exp Fatal Outcome/ or exp "Outcome Assessment (Health Care)"/ or exp "Outcome and Process Assessment (Health Care)"/ or ((patient outcome assessment or fatal outcome or outcome assessment or outcome) and process assessment).sh.
90. exp Chronic Disease/ or chronic disease.sh.
91. exp Survival Analysis/ or *Survival/ or exp Survival Rate/ or (survival analysis or *survival/ or survival rate).sh.
92. exp "Age of Onset"/ or age of onset.sh.
93. exp Terminal Care/ or terminal care.sh.
94. (disease control or disease course or adverse outcome or chronicity or deterioration or disease duration or disease exacerbation or general condition deterioration or general condition improvement or illness trajectory or remission or progress* or impairment or decline or failure or decrease or worsening or deterioration or degeneration).tw. or (disease control or disease course or adverse outcome or chronicity or deterioration or disease duration or disease exacerbation or general condition deterioration or general condition improvement or illness trajectory or remission or progress* or impairment or decline or failure or decrease or worsening or deterioration or degeneration).kf.
95. 88 or 89 or 90 or 91 or 92 or 93 or 94

Final combinations:

96. 11 and 32 and 95
97. 52 and 96
98. 68 and 96
99. 87 and 96
