# Supplementary material for: Comorbidity and progression of late onset Alzheimer’s disease: A systematic review
Source: PLoS One. 2017 May 4;12(5):e0177044. doi: 10.1371/journal.pone.0177044 (PMC5417646; doi:10.1371/journal.pone.0177044)
Supplement: S2 Appendix — (PDF) [file pone.0177044.s002.pdf]

S2 Appendix. Search strategy PsycINFO.

PsycINFO tools:
- id: key concepts
- ti: title
- ab: abstract
- sh: subject heading
- tw (ti+ab): text word

1) Alzheimer's Disease

1. exp Alzheimer's Disease/ or alzheimer*.sh.
2. (alzheimer sclerosis or alzheimer disease late onset or alzheimer type dementia or dementia primary senile degenerative or presenile alzheimer dementia or alzheimer syndrome or dementia alzheimer-type or senile dementia or dementia alzheimer or dementia alzheimer type or presenile dementia or dementia senile or late onset alzheimer disease or alzheimer's disease or primary senile degenerative dementia or syndrome alzheimer or dementia presenile or alzheimer disease assessment scale or alzheimer's disease or alzheimer's-disease or alzheimer-disease).tw. or (alzheimer sclerosis or alzheimer disease late onset or alzheimer type dementia or dementia primary senile degenerative or presenile alzheimer dementia or alzheimer syndrome or dementia alzheimer-type or senile dementia or dementia alzheimer or dementia alzheimer type or presenile dementia or dementia senile or late onset alzheimer disease or alzheimer's disease or primary senile degenerative dementia or syndrome alzheimer or dementia presenile or alzheimer disease assessment scale or alzheimer's disease or alzheimer's-disease or alzheimer-disease).id.
3. exp Dementia/ or dementia.sh.
4. (amentia* or dement* senile paranoid or dementia presenile or mental deterioration).tw. or (amentia* or dement* senile paranoid or dementia presenile or mental deterioration).id.
5. 1 or 2 or 3 or 4

2) Observational/Prognosis/Predictor/Comorbidity

6. Observational studies.ti. or epidemiologic studies/ or exp case-control studies/ or cross-sectional studies/
7. ((case adj3 control) or (cohort adj5 (study or studies or analy$)) or (follow-up adj5 (study or studies)) or (longitudinal or retrospective or prospective or (cross adj5 sectional)) or (observational adj5 (study or studies))).af.
8. 6 or 7
9. 5 and 8
10. exp Prognosis/ or prognos*.sh.
11. exp Probability/ or probab*.sh.
12. exp Prediction/ or predict*.sh.
13. exp Risk Factors/ or risk factor*.sh.
14. (decision support techniques or forecasting or predictive validity or predictor variable).tw. or (decision support techniques or forecasting or predictive validity or predictor variable).id.
15. ((risk adj prediction) or (predictor adj variabl??) or (increas* adj risk)).mp. or ((risk adj assessement?) or (predict* adj risk?) or (risk adj factor?) or (validat* or predict* or rule*)).tw. or (((risk adj prediction) or (predictor adj variabl??) or (increas* adj risk)).mp. or ((risk adj assessement?) or (predict* adj risk?) or (risk adj factor?) or (validat* or predict* or rule*)).id.)
16. ((predict* and (outcome* or risk* or model*)) or ((history or variable* or criteria or scor* or characteristic* or finding* or factor*) and (predict* or model* or decision* or identi* or prognos*)) or (decision* and (model* or clinical* or (logistic adj3 models)))).tw. or ((predict* and (outcome* or risk* or model*)) or ((history or variable* or criteria or scor* or characteristic* or finding* or factor*) and (predict* or model* or decision* or identi* or prognos*)) or (decision* and (model* or clinical* or (logistic adj3 models)))).id.
17. 10 or 11 or 12 or 13 or 14 or 15 or 16
18. exp Comorbidity/ or comorbidit*.sh.
19. (multimorbidit* or comorbidit*).tw. or (multimorbidit* or comorbidit*).id.
20. (co morbidit* or co-morbidit* or coomorbidit*).tw. or (co morbidit* or co-morbidit* or coomorbidit*).id.
21. (chronic diseases or cooccurring diseases or co-occurring diseases or co occurring diseases or clusters of diseases or disease burden or physical health or medical health or charlson or cumulative illness scale geriatrics or polymorbidity or disease count).tw. or (chronic diseases or cooccurring diseases or co-occurring diseases or co occurring diseases or clusters of diseases or disease burden or physical health or medical health or charlson or cumulative illness scale geriatrics or polymorbidity or disease count).id.
22. 18 or 19 or 20 or 21
23. 17 and 22

3) Multidimensional progression

24. exp Cognition/ or cognit*.sh.
25. exp Cognitions/
26. exp cognitive ability/ or cognitive ability.sh.
27. exp spatial ability/ or spatial ability.sh.
28. exp cognitive appraisal/ or cognitive appraisal.sh.
29. exp cognitive assessment/ or cognitive assessment.sh.
30. exp cognitive control/ or cognitive control.sh.

31. exp Cognitive Dissonance/ or cognitive dissonance.sh.
32. exp Cognitive Impairment/ or cognitive impairment.sh.
33. exp cognitive maps/ or wayfinding/ or direction perception/ or mental models/ or schema/ or spatial imagery/ or "spatial orientation (perception)"/ or (cognitive maps or wayfinding or direction perception or mental models or schema or spatial imagery or spatial orientation).sh.
34. exp cognitive processes/ or cognitive processes.sh.
35. cognit*.tw. or cognit*.id.
36. (consciousness disorders or overinclusion or cognitive performance or confusion or intellectual disability or perceptual disorders or mental competency or perception or thinking or aptitude or mild cognitive impairment or cognitive defect or cognitive generalization or cognitive complexity or cognitive contiguity or thought content or cognitive functioning or executive functioning or intellectual functioning or mathematical ability or reading ability or verbal ability or cognitive deficits or cognitive dysfunction or executive dysfunction or thought disturbances or human information process or cognitive science or information processing model or metacognition or intelligence or intelligence measures or intelligence quotient or expectations or irrational beliefs).tw. or (consciousness disorders or overinclusion or cognitive performance or confusion or intellectual disability or perceptual disorders or mental competency or perception or thinking or aptitude or mild cognitive impairment or cognitive defect or cognitive generalization or cognitive complexity or cognitive contiguity or thought content or cognitive functioning or executive functioning or intellectual functioning or mathematical ability or reading ability or verbal ability or cognitive deficits or cognitive dysfunction or executive dysfunction or thought disturbances or human information process or cognitive science or information processing model or metacognition or intelligence or intelligence measures or intelligence quotient or expectations or irrational beliefs).id.
37. exp Long Term Memory/ or exp Visuospatial Memory/ or exp False Memory/ or *Memory/ or exp Short Term Memory/ or exp Retrospective Memory/ or exp Visual Memory/ or exp Episodic Memory/ or exp Spatial Memory/ or exp Autobiographical Memory/ or exp Memory Disorders/ or exp Prospective Memory/ or exp Semantic Memory/ or exp Verbal Memory/ or (long term memory or visuospatial memory or false memory or *memory/ or short term memory or retrospective memory or visual memory or episodic memory or spatial memory or autobiographical memory or memory disorders or prospective memory or semantic memory or verbal memory).sh.
38. *Anterograde Amnesia/ or exp Amnesia/ or *Retrograde Amnesia/ or *Global Amnesia/ or (*anterograde amnesia/ or amnesia or *retrograde amnesia/ or *global amnesia/).sh.
39. exp memory decay/ or memory decay.sh.
40. exp memory trace/ or memory trace.sh.
41. (early memories or eidetic imagery or memory consolidation or reminiscence or "spontaneous recovery (learning)" or memory losses or memory disorders age related or retention disorder cognitive or memory disorder semantic or age-related memory disorder or remote memory or immediate memories or working memory or recall immediate or associative memory or auditory memory or olfactory memory or recognition or reference memory or repetition priming or sensory memory or tactile memory or memory bias or word list recall or word recognition).tw. or (early memories or eidetic imagery or memory consolidation or reminiscence or "spontaneous recovery (learning)" or memory losses or memory disorders age related or retention disorder cognitive or memory disorder semantic or age-related memory disorder or remote memory or immediate memories or working memory or recall immediate or associative memory or auditory memory or olfactory memory or recognition or reference memory or repetition priming or sensory memory or tactile memory or memory bias or word list recall or word recognition).id.
42. *Language/ or exp Speech Language Pathology/ or exp Language Disorders/ or (*language/ or speech language pathology or language disorders).sh.
43. exp Oral Communication/ or oral communication.sh.
44. exp Verbal Ability/ or exp Verbal Comprehension/ or exp Verbal Communication/ or (verbal ability or verbal comprehension or verbal communication).sh.
45. (speech-language pathology or verbal behavior or language disorder acquired or pathology speech or pathology language or verbal fluency or "speech and language" or linguistic or language ability or speech analysis or speech perception or speech disorder or language disability or language ability or language test or communication disorders or neurolinguistics).tw. or (speech-language pathology or verbal behavior or language disorder acquired or pathology speech or pathology language or verbal fluency or "speech and language" or linguistic or language ability or speech analysis or speech perception or speech disorder or language disability or language ability or language test or communication disorders or neurolinguistics).id.
46. exp Attention/ or attention.sh.
47. exp Problem Solving/ or problem solving.sh.
48. exp Decision Making/ or decision making.sh.
49. exp Reading/ or exp Reading Comprehension/ or exp Reading Ability/ or (reading or reading comprehension or reading ability).sh.
50. exp Perceptual Orientation/ or perceptual orientation.sh.
51. exp Judgment/ or exp Judgment Disturbances/ or (judgment or judgment disturbances).sh.
52. (executive function or executive control or concentration or shared decision making or mental speed or verbal reasoning or abstraction).tw. or (executive function or executive control or concentration or shared decision making or mental speed or verbal reasoning or abstraction).id.
53. 24 or 25 or 26 or 27 or 28 or 29 or 30 or 31 or 32 or 33 or 34 or 35 or 36 or 37 or 38 or 39 or 40 or 41 or 42 or 43 or 44 or 45 or 46 or 47 or 48 or 49 or 50 or 51 or 52

54. exp "activities of daily living"/ or activit* of daily living.sh.
55. exp Physical Activity/ or physical activit*.sh.
56. exp walking/ or exp motor performance/ or exp gait/ or (walking or motor performance or gait).sh.

57. exp Physical Strength/ or physical strength.sh.
58. exp Self Management/ or exp Self Care Skills/ or (self management or self care skills).sh.
59. exp Locomotion/ or locomot*.sh.
60. exp Equilibrium/ or exp Posture/ or exp Falls/ or exp Motor Coordination/ or exp Exercise/ or exp Motor Processes/ or exp Perceptual Motor Processes/ or (equilibrium or posture or falls or motor coordination or exercise or motor processes or perceptual motor processes).sh.
61. exp Physical Activity/ or exp Physical Disorders/ or exp Physical Endurance/ or exp Physical Health/ or exp Physical Mobility/ or exp Physical Agility/ or (physical activity or physical disorders or physical endurance or physical health or physical mobility or physical agility).sh.
62. exp Motor Performance/ or exp Motor Skills/ or (motor performance or motor skills).sh.
63. exp Physical Strength/ or physical strength.sh.
64. (hand strength or gait disorders or compressive strength or muscle strength or accidental falls or personal autonomy or work capacity evaluation or physical function or ambulation or limitation of activity chronic or self-care or self-management or musculoskeletal equilibrium or postural equilibrium or grasps or grips or pinch strength).tw. or (hand strength or gait disorders or compressive strength or muscle strength or accidental falls or personal autonomy or work capacity evaluation or physical function or ambulation or limitation of activity chronic or self-care or self-management or musculoskeletal equilibrium or postural equilibrium or grasps or grips or pinch strength).id.
65. 54 or 55 or 56 or 57 or 58 or 59 or 60 or 61 or 62 or 63 or 64

66. exp Mental Disorders/ or mental disorder*.sh.
67. exp Geriatric Psychiatry/ or *Psychiatry/ or geriatr* psychiat*.sh. or psychiatr*.sh.
68. exp Behavior Disorders/ or exp Behavior Modification/ or *Behavior/ or exp Behavior Problems/ or exp Behavior Change/ or exp Aggressive Behavior/ or exp Illness Behavior/ or (behavior disorders or behavior modification or *behavior/ or behavior problems or behavior change or aggressive behavior or illness behavior).sh.
69. exp Anxiety Disorders/ or exp Anxiety/ or (anxiety or anxiety disorder*).sh.
70. exp Delirium/ or exp Mental Confusion/ or (delirium or mental confusion).sh.
71. exp Emotions/ or emotion*.sh.
72. exp Emotional States/ or emotional state*.sh.
73. exp Aggressiveness/ or exp Aggressive Behavior/ or (aggressiveness or aggressive behavior).sh.
74. exp Hostility/ or hostility.sh.
75. exp Delusions/ or delusion*.sh.
76. exp Visual Hallucinations/ or exp Auditory Hallucinations/ or exp Hallucinations/ or (visual hallucinations or auditory hallucinations or hallucinations).sh.
77. exp Agitation/ or agitation.sh.
78. exp Major Depression/ or major depression.sh.
79. exp Euphoria/ or euphoria.sh.
80. exp Apathy/ or apathy.sh.
81. exp Irritability/ or irritability.sh.
82. (emotional disorder or mental patient or mental instability or mood disorder or organic psychosyndrome or dysphoria or neuropsychological assessment or psychiatric evaluation or psychiatric disorders or indifference or disinhibition or lability or aberrant motor behavior or hallucinat* or agitat* or anxiety elation or apath* or neuropsychiat*).tw. or (emotional disorder or mental patient or mental instability or mood disorder or organic psychosyndrome or dysphoria or neuropsychological assessment or psychiatric evaluation or psychiatric disorders or indifference or disinhibition or lability or aberrant motor behavior or hallucinat* or agitat* or anxiety elation or apath* or neuropsychiat*).id.
83. 66 or 67 or 68 or 69 or 70 or 71 or 72 or 73 or 74 or 75 or 76 or 77 or 78 or 79 or 80 or 81 or 82

84. exp Disease Course/ or disease course.sh.
85. exp Chronic Mental Illness/ or exp Chronic Illness/ or (chronic mental illness or chronic illness).sh.
86. exp "Onset (Disorders)"/ or onset disorder*.sh.
87. (disease progression or patient outcome assessment or fatal outcome or outcome assessment or process assessment or survival analysis or survival or survival rate or age of onset or terminal care or disease control or disease course or adverse outcome or chronicity or deterioration or disease duration or disease exacerbation or general condition deterioration or general condition improvement or illness trajectory or remission or progress* or impairment or decline or failure or decrease or worsening or deterioration or degeneration).tw. or (disease progression or patient outcome assessment or fatal outcome or outcome assessment or process assessment or survival analysis or survival or survival rate or age of onset or terminal care or disease control or disease course or adverse outcome or chronicity or deterioration or disease duration or disease exacerbation or general condition deterioration or general condition improvement or illness trajectory or remission or progress* or impairment or decline or failure or decrease or worsening or deterioration or degeneration).id.
88. 84 or 85 or 86 or 87

Final combinations:

89. 9 and 22 and 88
90. 53 and 89
91. 65 and 89
92. 83 and 89
