# Supplementary material for: Comorbidity and progression of late onset Alzheimer’s disease: A systematic review
Source: PLoS One. 2017 May 4;12(5):e0177044. doi: 10.1371/journal.pone.0177044 (PMC5417646; doi:10.1371/journal.pone.0177044)
Supplement: S3 Appendix — (PDF) [file pone.0177044.s003.pdf]

S3 Appendix. Search strategy EMBASE.

EMBASE tools:
- kw: keyword
- ti: title
- ab: abstract
- tw (ti+ab): text word

1) Alzheimer's Disease

1. exp Alzheimer Disease/ or alzheimer*.sh.
2. (alzheimer sclerosis or alzheimer disease late onset or alzheimer type dementia or dementia primary senile degenerative or presenile alzheimer dementia or alzheimer syndrome or dementia alzheimer-type or senile dementia or dementia alzheimer or dementia alzheimer type or presenile dementia or dementia senile or late onset alzheimer disease or alzheimer's disease or primary senile degenerative dementia or syndrome alzheimer or dementia presenile or alzheimer disease assessment scale or alzheimer's disease or alzheimer's-disease or alzheimer-disease).tw. or (alzheimer sclerosis or alzheimer disease late onset or alzheimer type dementia or dementia primary senile degenerative or presenile alzheimer dementia or alzheimer syndrome or dementia alzheimer-type or senile dementia or dementia alzheimer or dementia alzheimer type or presenile dementia or dementia senile or late onset alzheimer disease or alzheimer's disease or primary senile degenerative dementia or syndrome alzheimer or dementia presenile or alzheimer disease assessment scale or alzheimer's disease or alzheimer's-disease or alzheimer-disease).kw.
3. exp Dementia/ or dementia.sh.
4. exp dementia assessment/ or exp presenile dementia/ or exp senile dementia/ or (dementia assessment or presenile dementia or senile dementia).sh.
5. (amentia* or dement* senile paranoid or dementia presenile or mental deterioration).tw. or (amentia* or dement* senile paranoid or dementia presenile or mental deterioration).kw.
6. 1 or 2 or 3 or 4 or 5

2) Observational/Prognosis/Predictor/Comorbidity

7. "Observational studies".ti. or epidemiologic studies/ or exp case-control studies/ or cross-sectional studies/
8. ((case adj3 control) or (cohort adj5 (study or studies or analy$)) or (follow-up adj5 (study or studies)) or (longitudinal or retrospective or prospective or (cross adj5 sectional)) or (observational adj5 (study or studies))).af.
9. 7 or 8
10. 6 and 9
11. exp Probability/ or probab*.sh.
12. exp Prediction/ or predict*.sh.
13. exp risk factor/ or risk factor*.sh.
14. exp Prognosis/ or prognos*.sh.
15. exp "prediction and forecasting"/ or exp forecasting/ or ((prediction and forecasting) or forecasting).sh.
16. (decision support techniques or prediction or predictive validity or predictor variable).tw. or (decision support techniques or prediction or predictive validity or predictor variable).kw.
17. ((risk adj prediction) or (predictor adj variabl??) or (increas* adj risk)).mp. or ((risk adj assessement?) or (predict* adj risk?) or (risk adj factor?) or (validat* or predict* or rule*)).tw. or (((risk adj prediction) or (predictor adj variabl??) or (increas* adj risk)).mp. or ((risk adj assessement?) or (predict* adj risk?) or (risk adj factor?) or (validat* or predict* or rule*)).kw.)
18. ((predict* and (outcome* or risk* or model*)) or ((history or variable* or criteria or scor* or characteristic* or finding* or factor*) and (predict* or model* or decision* or identi* or prognos*)) or (decision* and (model* or clinical* or (logistic adj3 models)))).tw. or ((predict* and (outcome* or risk* or model*)) or ((history or variable* or criteria or scor* or characteristic* or finding* or factor*) and (predict* or model* or decision* or identi* or prognos*)) or (decision* and (model* or clinical* or (logistic adj3 models)))).kw.
19. 11 or 12 or 13 or 14 or 15 or 16 or 17 or 18
20. exp Comorbidity/ or comorbidit*.sh.
21. exp comorbidity assessment/ or comorbidity assessment.sh.
22. (multimorbidit* or comorbidit*).tw. or (multimorbidit* or comorbidit*).kw.
23. (co morbidit* or co-morbidit* or coomorbidit*).tw. or (co morbidit* or co-morbidit* or coomorbidit*).kw.
24. (chronic diseases or cooccurring diseases or co-occurring diseases or co occurring diseases or clusters of diseases or disease burden or physical health or medical health or charlson or cumulative illness scale geriatrics or polymorbidity or disease count).tw. or (chronic diseases or cooccurring diseases or co-occurring diseases or co occurring diseases or clusters of diseases or disease burden or physical health or medical health or charlson or cumulative illness scale geriatrics or polymorbidity or disease count).kw.
25. 20 or 21 or 22 or 23 or 24
26. 19 and 25

3) Multidimensional progression

27. exp Cognition/ or cognit*.sh.
28. exp mild cognitive impairment/ or exp cognitive defect/ or (mild cognitive impairment or cognitive defect).sh.
29. exp cognition assessment/ or cognitive assessment.sh.
30. cognit*.tw. or cognit*.kw.

31. (consciousness disorders or overinclusion or cognitive performance or confusion or intellectual disability or perceptual disorders or mental competency or perception or thinking or aptitude or mild cognitive impairment or cognitive defect or cognitive generalization or cognitive complexity or cognitive contiguity or cognitive dissonance or cognitive appraisal or cognitive maps or wayfinding or spatial imagery or direction perception or thought content or cognitive functioning or executive functioning or intellectual functioning or mathematical ability or reading ability or verbal ability or cognitive deficits or cognitive dysfunction or executive dysfunction or thought disturbances or human information process or cognitive science or information processing model or metacognition or intelligence or intelligence measures or intelligence quotient or cognitive disorders).tw. or (consciousness disorders or overinclusion or cognitive performance or confusion or intellectual disability or perceptual disorders or mental competency or perception or thinking or aptitude or mild cognitive impairment or cognitive defect or cognitive generalization or cognitive complexity or cognitive contiguity or cognitive dissonance or cognitive appraisal or cognitive maps or wayfinding or spatial imagery or direction perception or thought content or cognitive functioning or executive functioning or intellectual functioning or mathematical ability or reading ability or verbal ability or cognitive deficits or cognitive dysfunction or executive dysfunction or thought disturbances or human information process or cognitive science or information processing model or metacognition or intelligence or intelligence measures or intelligence quotient or cognitive disorders).kw.
32. exp memory/ or memory.sh.
33. exp short term memory/ or exp sensory memory/ or exp spatial memory test/ or exp explicit memory/ or exp memory disorder/ or exp memory bias/ or exp associative memory/ or exp false memory/ or exp episodic memory/ or exp auditory memory/ or exp autobiographical memory/ or exp retrospective memory/ or exp verbal memory/ or exp working memory/ or exp implicit memory/ or exp prospective memory/ or exp memory assessment/ or exp memory consolidation/ or exp long term memory/ or exp olfactory memory/ or exp spatial memory/ or exp visual memory/ or exp tactile memory/ or exp reference memory/ or exp semantic memory/ or (short term memory or sensory memory or spatial memory test or explicit memory or memory disorder or memory bias or associative memory or false memory or episodic memory or auditory memory or autobiographical memory or retrospective memory or verbal memory or working memory or implicit memory or prospective memory or memory assessment or memory consolidation or long term memory or olfactory memory or spatial memory or visual memory or tactile memory or reference memory or semantic memory).sh.
34. (spatial memory disorders or memory disorder or memory losses or memory disorders age related or retention disorder cognitive or memory disorder semantic or age-related memory disorder or memory deficits or spatial memory disorder or cognitive retention disorder or immediate memories or working memory or memory shortterm or recall immediate or amnesia or anterograde amnesia or global amnesia or retrograde amnesia or memory decay or memory trace or visuospatial memory or associative memory or auditory memory or false memory or olfactory memory or recognition or reference memory or repetition priming or retrospective memory or sensory memory or tactile memory or memory bias or word list recall or word recognition or working memory).tw. or (spatial memory disorders or memory disorder or memory losses or memory disorders age related or retention disorder cognitive or memory disorder semantic or age-related memory disorder or memory deficits or spatial memory disorder or cognitive retention disorder or immediate memories or working memory or memory shortterm or recall immediate or amnesia or anterograde amnesia or global amnesia or retrograde amnesia or memory decay or memory trace or visuospatial memory or associative memory or auditory memory or false memory or olfactory memory or recognition or reference memory or repetition priming or retrospective memory or sensory memory or tactile memory or memory bias or word list recall or word recognition or working memory).kw.
35. exp language/ or language.sh.
36. exp speech/ or speech.sh.
37. exp speech language pathologist/ or exp language ability/ or exp language disability/ or exp "speech and language"/ or exp "speech and language assessment"/ or ((((speech language pathologist or language ability or language disability or speech) and language) or speech) and language assessment).sh.
38. exp speech disorder/ or exp speech analysis/ or exp speech perception/ or (speech disorder or speech analysis or speech perception).sh.
39. (language disorders or language disorder acquired or pathology speech or pathology language or verbal fluency or linguistic or oral communication or communication disorders).tw. or (language disorders or language disorder acquired or pathology speech or pathology language or verbal fluency or linguistic or oral communication or communication disorders).kw.
40. exp Verbal Behavior/ or verbal behavior.sh.
41. exp Executive Function/ or executive function*.sh.
42. exp Attention/ or attention.sh.
43. exp Problem Solving/ or problem solving.sh.
44. exp Decision Making/ or decision making.sh.
45. exp Orientation/ or orientation.sh.
46. exp Reading/ or reading.sh.
47. exp Judgment/ or judgment.sh.
48. exp mental concentration/ or mental concentration.sh.
49. exp mental capacity/ or mental capacity.sh.
50. exp aptitude/ or aptitude.sh.
51. (executive control or concentration or shared decision making or mental speed or verbal reasoning or abstraction).tw. or (executive control or concentration or shared decision making or mental speed or verbal reasoning or abstraction).kw.
52. 27 or 28 or 29 or 30 or 31 or 32 or 33 or 34 or 35 or 36 or 37 or 38 or 39 or 40 or 41 or 42 or 43 or 44 or 45 or 46 or 47 or 48 or 49 or 50 or 51

53. exp daily life activity/ or daily life activity.sh.

54. exp physical mobility/ or exp physical capacity/ or exp physical performance/ or exp physical activity/ or exp physical disability/ or (physical mobility or physical capacity or physical performance or physical activity or physical disability).sh.
55. exp motor coordination/ or exp motor control/ or exp motor dysfunction/ or exp motor performance/ or exp motor activity/ or (motor coordination or motor control or motor dysfunction or motor performance or motor activity).sh.
56. exp Locomotion/ or locomot*.sh.
57. exp walking/ or exp walking speed/ or exp walking difficulty/ or (walking or walking speed or walking difficulty).sh.
58. exp gait/ or exp unsteady gait/ or exp gait disorder/ or (gait or unsteady gait or gait disorder).sh.
59. exp endurance/ or endurance.sh.
60. exp balance disorder/ or exp balance impairment/ or (balance disorder or balance impairment).sh.
61. exp body equilibrium/ or body equilibrium.sh.
62. exp falling/ or falling.sh.
63. exp grip strength/ or exp pinch strength/ or exp hand strength/ or exp muscle strength/ or exp grip strength test/ or exp strength/ or (grip strength or pinch strength or hand strength or muscle strength or grip strength test or strength).sh.
64. exp Self Care/ or self care.sh.
65. exp personal autonomy/ or exp personal hygiene/ or (personal autonomy or personal hygiene).sh.
66. exp work disability/ or exp work capacity/ or (work disability or work capacity).sh.
67. (physical function or ambulation or limitation of activity chronic or self management or self-care or self-management or musculoskeletal equilibrium or postural equilibrium or grasps or grips or pinch strength or (physical function or ambulation or limitation of activity chronic or self management or self-care or self-management or musculoskeletal equilibrium or postural equilibrium or grasps or grips or pinch strength) or (physical function or ambulation or limitation of activity chronic or self management or self-care or self-management or musculoskeletal equilibrium or postural equilibrium or grasps or grips or pinch strength)).tw. or (physical function or ambulation or limitation of activity chronic or self management or self-care or self-management or musculoskeletal equilibrium or postural equilibrium or grasps or grips or pinch strength).kw.
68. 53 or 54 or 55 or 56 or 57 or 58 or 59 or 60 or 61 or 62 or 63 or 64 or 65 or 66 or 67

69. exp mental instability/ or mental instability.sh.
70. exp psychiatry/ or psychiatr*.sh.
71. exp gerontopsychiatry/ or gerontopsychiatr*.sh.
72. exp neuropsychiatry/ or neuropsychiat*.sh.
73. exp behavior control/ or exp illness behavior/ or exp behavior disorder assessment/ or exp behavior assessment/ or exp behavior change/ or exp behavior modification/ or exp behavior disorder/ or (behavior control or illness behavior or behavior disorder assessment or behavior assessment or behavior change or behavior modification or behavior disorder).sh.
74. exp anxiety assessment/ or exp Depression Anxiety Stress Scale/ or exp anxiety/ or exp anxiety disorder/ or exp generalized anxiety disorder/ or exp "mixed anxiety and depression"/ or ((anxiety assessment or Depression Anxiety Stress Scale or anxiety or anxiety disorder or generalized anxiety disorder or mixed anxiety) and depression).sh.
75. exp confusion/ or confusion.sh.
76. exp delirium/ or delirium.sh.
77. exp emotional disorder/ or emotional disorder.sh.
78. exp mood disorder assessment/ or exp mood change/ or exp mood disorder/ or (mood disorder assessment or mood change or mood disorder).sh.
79. exp organic psychosyndrome/ or organic psychosyndrome.sh.
80. exp aggression/ or exp hostility/ or (aggression or hostility).sh.
81. exp anger/ or anger.sh.
82. exp Apathy/ or apathy.sh.
83. exp Depression/ or depression.sh.
84. exp dysphoria/ or dysphoria.sh.
85. exp delusion/ or delusion.sh.
86. exp auditory hallucination/ or exp hallucination/ or exp visual hallucination/ or (auditory hallucination or hallucination or visual hallucination).sh.
87. exp agitation/ or exp agitation assessment/ or (agitation or agitation assessment).sh.
88. exp Euphoria/ or euphoria.sh.
89. exp Irritability/ or irritability.sh.
90. (mental patient or mental disorder or neuropsychological assessment or psychiatric evaluation or psychiatric disorders or indifference or disinhibition or lability or aberrant motor behavior or hallucinat* or agitat* or anxiety elation or apath* or neuropsychiat*).tw. or (mental patient or mental disorder or neuropsychological assessment or psychiatric evaluation or psychiatric disorders or indifference or disinhibition or lability or aberrant motor behavior or hallucinat* or agitat* or anxiety elation or apath* or neuropsychiat*).kw.
91. 69 or 70 or 71 or 72 or 73 or 74 or 75 or 76 or 77 or 78 or 79 or 80 or 81 or 82 or 83 or 84 or 85 or 86 or 87 or 88 or 89 or 90

92. exp Disease Course/ or disease course.sh.
93. exp disease control/ or disease control.sh.
94. exp outcome assessment/ or exp adverse outcome/ or (outcome assessment or adverse outcome).sh.
95. exp chronic disease/ or exp chronic patient/ or (chronic disease or chronic patient).sh.
96. exp chronicity/ or chronicity.sh.
97. exp survival rate/ or exp survival/ or exp survival prediction/ or (survival rate or survival or survival prediction).sh.
98. exp onset age/ or onset age.sh.

99. exp Terminal Care/ or terminal care.sh.
100. exp disease duration/ or disease duration.sh.
101. exp general condition deterioration/ or exp deterioration/ or exp mental deterioration/ or (general condition deterioration or deterioration or mental deterioration).sh.
102. exp disease exacerbation/ or disease exacerbation.sh.
103. exp general condition improvement/ or general condition improvement.sh.
104. exp illness trajectory/ or illness trajectory.sh.
105. (disease control or disease progression or remission or progress* or impairment or decline or failure or decrease or worsening or deterioration or degeneration).tw. or (disease control or disease progression or remission or progress* or impairment or decline or failure or decrease or worsening or deterioration or degeneration).kw.
106. 92 or 93 or 94 or 95 or 96 or 97 or 98 or 99 or 100 or 101 or 102 or 103 or 104 or 105

Final combinations:

107. 10 and 25 and 106
108. 52 and 107
109. 68 and 107
110. 91 and 107
