# Supplementary material for: Comorbidity and progression of late onset Alzheimer’s disease: A systematic review
Source: PLoS One. 2017 May 4;12(5):e0177044. doi: 10.1371/journal.pone.0177044 (PMC5417646; doi:10.1371/journal.pone.0177044)
Supplement: S4 Appendix — (PDF) [file pone.0177044.s004.pdf]

S4 Appendix. Search strategy Cochrane.

1) Alzheimer's Disease

#1 MeSH descriptor: [Alzheimer Disease] explode all trees

#2 alzheimer sclerosis or alzheimer disease late onset or alzheimer type dementia or dementia primary senile degenerative or presenile alzheimer dementia or alzheimer syndrome or dementia alzheimer-type or senile dementia or dementia alzheimer or dementia alzheimer type or presenile dementia or dementia senile or late onset alzheimer disease or alzheimer's disease or primary senile degenerative dementia or syndrome alzheimer or dementia presenile or alzheimer disease assessment scale or alzheimer's disease or alzheimer's-disease or alzheimer-disease

#3 MeSH descriptor: [Dementia] explode all trees

#4 amentia* or dement* senile paranoid or dementia presenile or mental deterioration

#5 #1 or #2 or #3 or #4

2) Observational/Prognosis/Predictor/Comorbidity

#6 MeSH descriptor: [Patients] explode all trees

#7 Observational studies or epidemiologic studies or exp case-control studies or cross-sectional studies or (case adj3 control) or (cohort adj5 (study or studies or analy$)) or (follow-up adj5 (study or studies)) or (longitudinal or retrospective or prospective or (cross adj5 sectional)) or (observational adj5 (study or studies))

#8 #6 or #7

#9 #5 and #8

#10 MeSH descriptor: [Prognosis] explode all trees

#11 MeSH descriptor: [Probability] explode all trees

#12 MeSH descriptor: [Forecasting] explode all trees

#13 MeSH descriptor: [Risk Factors] explode all trees

#14 MeSH descriptor: [Decision Support Techniques] explode all trees

#15 prediction or predictive validity or predictor variable

#16 (risk adj prediction) or (predictor adj variabl??) or (increas* adj risk) or (risk adj assessement?) or (predict* adj risk?) or (risk adj factor?) or (validat* or predict* or rule*)

#17 ((predict* and (outcome* or risk* or model*)) or ((history or variable* or criteria or scor* or characteristic* or finding* or factor*) and (predict* or model* or decision* or identi* or prognos*)) or (decision* and (model* or clinical* or (logistic adj3 models))))

#18 #10 or #11 or #12 or #13 or #14 or #15 or #16 or #17

#19 MeSH descriptor: [Comorbidity] explode all trees

#20 MeSH descriptor: [Chronic Disease] explode all trees

#21 (multimorbidit* or comorbidit*)

#22 (co morbidit* or co-morbidit* or coomorbidit*)

#23 (cooccurring diseases or co-occurring diseases or co occurring diseases or clusters of diseases or comorbidity assessment or disease burden or physical health or medical health or charlson or cumulative illness scale geriatrics or polymorbidity or disease count)

#24 #19 or #20 or #21 or #22 or #23

#25 #18 and #24

3) **Multidimensional progression**

#26 MeSH descriptor: [Cognition] explode all trees

#27 MeSH descriptor: [Cognition Disorders] explode all trees

#28 MeSH descriptor: [Confusion] explode all trees

#29 MeSH descriptor: [Consciousness Disorders] explode all trees

#30 MeSH descriptor: [Intellectual Disability] explode all trees

#31 MeSH descriptor: [Perception] explode all trees

#32 MeSH descriptor: [Perceptual Disorders] explode all trees

#33 MeSH descriptor: [Mental Competency] explode all trees

#34 overinclusion or cognitive performance or thinking or aptitude or mild cognitive impairment or cognitive defect or cognitive generalization or cognitive complexity or cognitive contiguity or cognitive dissonance or cognitive appraisal or cognitive maps or wayfinding or spatial imagery or direction perception or thought content or cognitive functioning or executive functioning or intellectual functioning or mathematical ability or reading ability or verbal ability or cognitive deficits or cognitive dysfunction or executive dysfunction or thought disturbances or human information process or cognitive science or information processing model or metacognition or intelligence or intelligence measures or intelligence quotient or cognitive disorders or mild cognitive impairment or cognitive defect or cognition assessment

#35 cognit*

#36 MeSH descriptor: [Aptitude] explode all trees

#37 MeSH descriptor: [Thinking] explode all trees

#38 MeSH descriptor: [Attention] explode all trees

#39 MeSH descriptor: [Orientation] explode all trees

#40 MeSH descriptor: [Memory] explode all trees

#41 MeSH descriptor: [Memory Disorders] explode all trees

#42 short term memory or sensory memory or spatial memory test or explicit memory or memory bias or associative memory or false memory or episodic memory or auditory memory or autobiographical memory or retrospective memory or verbal memory or working memory or implicit memory or prospective memory or memory assessment or memory consolidation or long term memory or olfactory memory or spatial memory or visual memory or tactile memory or reference memory or semantic memory or spatial memory disorders or memory losses or memory disorders age related or retention disorder cognitive or memory disorder semantic or age-related memory disorder or memory deficits or spatial memory disorder or cognitive retention disorder or immediate memories or working memory or memory shortterm or recall immediate or amnesia or anterograde amnesia or global amnesia or retrograde amnesia or memory decay or memory trace or visuospatial memory or associative memory or auditory memory or false memory or olfactory memory or recognition or reference memory or repetition priming or retrospective memory or sensory memory or memory bias or word list recall or word recognition or working memory
#43 MeSH descriptor: [Language] explode all trees
#44 MeSH descriptor: [Language Disorders] explode all trees
#45 MeSH descriptor: [Speech] explode all trees
#46 MeSH descriptor: [Speech Disorders] explode all trees
#47 MeSH descriptor: [Communication Disorders] explode all trees
#48 speech language pathologist or language ability or language disability or "speech and language" or "speech and language assessment" or speech analysis or speech perception or language disorder acquired or pathology speech or pathology language or verbal fluency or linguistic or oral communication
#49 MeSH descriptor: [Verbal Behavior] explode all trees
#50 MeSH descriptor: [Executive Function] explode all trees
#51 MeSH descriptor: [Decision Making] explode all trees
#52 MeSH descriptor: [Problem Solving] explode all trees
#53 MeSH descriptor: [Reading] explode all trees
#54 MeSH descriptor: [Judgment] explode all trees
#55 executive control or concentration or shared decision making or mental speed or verbal reasoning or abstraction
#56 #26 or #27 or #28 or #29 or #30 or #31 or #32 or #33 or #34 or #35 or #36 or #37 or #38 or #39 or #40 or #41 or #42 or #43 or #44 or #45 or #46 or #47 or #48 or #49 or #50 or #51 or #52 or #53 or #54 or #55

#57 MeSH descriptor: [Activities of Daily Living] explode all trees
#58 MeSH descriptor: [Exercise] explode all trees
#59 MeSH descriptor: [Motor Activity] explode all trees
#60 MeSH descriptor: [Physical Endurance] explode all trees
#61 MeSH descriptor: [Motor Skills Disorders] explode all trees
#62 MeSH descriptor: [Locomotion] explode all trees
#63 MeSH descriptor: [Gait Disorders, Neurologic] explode all trees
#64 MeSH descriptor: [Walking] explode all trees
#65 MeSH descriptor: [Psychomotor Disorders] explode all trees
#66 MeSH descriptor: [Movement Disorders] explode all trees
#67 MeSH descriptor: [Accidental Falls] explode all trees
#68 MeSH descriptor: [Postural Balance] explode all trees
#69 MeSH descriptor: [Muscle Strength] explode all trees
#70 MeSH descriptor: [Pinch Strength] explode all trees
#71 MeSH descriptor: [Hand Strength] explode all trees
#72 MeSH descriptor: [Compressive Strength] explode all trees
#73 MeSH descriptor: [Work Capacity Evaluation] explode all trees
#74 MeSH descriptor: [Self Care] explode all trees
#75 MeSH descriptor: [Personal Autonomy] explode all trees
#76 living assessment or lifting effort or weight bearing or weight lifting or writing or physical inactivity or physical performance or physical capacity or exercise tolerance or motor performance or object manipulation or self help or self concept or self control or personal hygiene or personal needs or psychomotor activity or driving ability or gesture or handedness or handwriting or self stimulation or skill or psychomotor disorder or unsteady gait or motor dysfunction or motor coordination or motor dysfunction assessment or motor control or body equilibrium or fall risk or fall risk assessment or falling or ability level or activity level or assisted living or daily activities or habilitation or independent living programs or physical mobility or self care skills or physical function or ambulation or limitation of activity chronic or self management or self-care or self-management or musculoskeletal equilibrium or equilibrium postural or grasp*
#77 #57 or #58 or #59 or #60 or #61 or #62 or #63 or #64 or #65 or #66 or #67 or #68 or #69 or #70 or #71 or #72 or #73 or #74 or #75 or #76

#78 MeSH descriptor: [Neuropsychiatry] explode all trees
#79 MeSH descriptor: [Neurobehavioral Manifestations] explode all trees
#80 MeSH descriptor: [Mental Disorders] explode all trees
#81 MeSH descriptor: [Behavior Control] explode all trees
#82 MeSH descriptor: [Behavioral Symptoms] explode all trees
#83 MeSH descriptor: [Affective Symptoms] explode all trees
#84 MeSH descriptor: [Mood Disorders] explode all trees
#85 MeSH descriptor: [Psychiatry] explode all trees

#86 MeSH descriptor: [Geriatric Psychiatry] explode all trees
#87 MeSH descriptor: [Neuropsychiatry] explode all trees
#88 MeSH descriptor: [Anxiety Disorders] explode all trees
#89 MeSH descriptor: [Confusion] explode all trees
#90 MeSH descriptor: [Delirium] explode all trees
#91 MeSH descriptor: [Aggression] explode all trees
#92 MeSH descriptor: [Irritable Mood] explode all trees
#93 MeSH descriptor: [Hostility] explode all trees
#94 MeSH descriptor: [Anger] explode all trees
#95 MeSH descriptor: [Apathy] explode all trees
#96 MeSH descriptor: [Depression] explode all trees
#97 MeSH descriptor: [Delusions] explode all trees
#98 MeSH descriptor: [Hallucinations] explode all trees
#99 MeSH descriptor: [Psychomotor Agitation] explode all trees
#100 mental patient or mental disorder or neuropsychological assessment or psychiatric evaluation or psychiatric disorders or indifference or disinhibition or lability or aberrant motor behavior or hallucinat* or agitat* or dysphoria or anxiety elation or apath* or neuropsychiat*
#101 #78 or #79 or #80 or #81 or #82 or #83 or #84 or #85 or #86 or #87 or #88 or #89 or #90 or #91 or #92 or #93 or #94 or #95 or #96 or #97 or #98 or #99 or #100

#102 MeSH descriptor: [Disease Progression] explode all trees
#103 MeSH descriptor: [Outcome and Process Assessment (Health Care)] explode all trees
#104 MeSH descriptor: [Patient Outcome Assessment] explode all trees
#105 MeSH descriptor: [Survival Analysis] explode all trees
#106 MeSH descriptor: [Age of Onset] explode all trees
#107 MeSH descriptor: [Chronic Disease] explode all trees
#108 disease course or disease control or adverse outcome or chronic patient or chronicity or survival rate or survival or survival prediction or terminal care or disease duration or general condition deterioration or deterioration or mental deterioration or disease exacerbation or general condition improvement or illness trajectory or remission or progress* or impairment or decline or failure or decrease or worsening or deterioration or degeneration
#109 #102 or #103 or #104 or #105 or #106 or #107 or #108

Final combinations:

#110 #9 and #25 and #109
#111 #110 and #56
#112 #110 and #77
#113 #110 and #101
