# Supplementary material for: Comorbidity and progression of late onset Alzheimer’s disease: A systematic review
Source: PLoS One. 2017 May 4;12(5):e0177044. doi: 10.1371/journal.pone.0177044 (PMC5417646; doi:10.1371/journal.pone.0177044)
Supplement: S5 Appendix — (PDF) [file pone.0177044.s005.pdf]

S5 Appendix. Quality Assessment.

Author (year)	Representative inclusion criteria	Comparable control group	Independent assessment of outcome	Independent assessment of comorbidity	Correction for age	Correction for education*	Correction for baseline progression	Follow up > 1 year	Follow-up rate > 60% at end of study	Total score
**Lyketsos et al. (1999)**	+	+	+	+	?	NA	NA	NA	NA	80.00%
**Tekin et al. (2001)**	+	+	+	?	+	-	NA	NA	NA	66.67%
**Doraiswamy et al. (2002)**	+	-	+	+	+	+	NA	NA	NA	83.33%
**Formiga et al. (2009)**	+	+	+	+	+	?	NA	NA	NA	83.33%
**Oosterveld et al. (2014)**	+	+	+	+	+	-	NA	NA	NA	83.33%
**Aguero-Torres et al. (1998)**	+	+	+	+	+	+	+	+	-	88.89%
**Boksay et al. (2005)**	-	+	+	?	?	-	?	+	+	44.44%
**Solomon et al. (2011)**	+	+	+	+	+	+	+	+	+	100.00%
**Leoutsakos et al. (2012)**	+	+	+	+	+	+	+	+	-	88.89%
**Melis et al. (2013)**	+	+	+	+	+	+	+	+	+	100.00%
**Aubert et al. (2015)**	+	+	+	?	+	-	+	+	+	77.78%

NA = not applicable

* correction for education was only rated for studies examining cognition
