# Supplementary material for: Comorbidity and progression of late onset Alzheimer’s disease: A systematic review
Source: PLoS One. 2017 May 4;12(5):e0177044. doi: 10.1371/journal.pone.0177044 (PMC5417646; doi:10.1371/journal.pone.0177044)
Supplement: S6 Appendix — (PDF) [file pone.0177044.s006.pdf]

S6 Appendix. PRISMA Checklist.

Section/topic	#	Checklist item	Reported on page #
**TITLE**			
Title	1	Identify the report as a systematic review, meta-analysis, or both.<br>*Comorbidity and Progression of Late Onset Alzheimer's Disease: A Systematic Review*	1
ABSTRACT			
Structured summary	2	Provide a structured summary including, as applicable: background; objectives; data sources; study eligibility criteria, participants, and interventions; study appraisal and synthesis methods; results; limitations; conclusions and implications of key findings; systematic review registration number.	2
INTRODUCTION			
Rationale	3	Describe the rationale for the review in the context of what is already known.<br>*"Knowing which factors are associated with decline would be useful for understanding disease progression, as well as for secondary prevention and individual prognosis."*	3
Objectives	4	Provide an explicit statement of questions being addressed with reference to participants, interventions, comparisons, outcomes, and study design (PICOS).<br>*"This review investigates whether there is evidence for an association between comorbid disease burden and cognitive, functional and psychiatric symptoms in individuals with LOAD, both cross-sectionally and longitudinally."*	3
METHODS			
Protocol and registration	5	Indicate if a review protocol exists, if and where it can be accessed (e.g., Web address), and, if available, provide registration information including registration number.<br>*"The protocol of this review was registered with PROSPERO and can be accessed through DIO: 10.15124/CRD42015027046."*	4
Eligibility criteria	6	Specify study characteristics (e.g., PICOS, length of follow-up) and report characteristics (e.g., years considered, language, publication status) used as criteria for eligibility, giving rationale.<br>*"In order to meet the inclusion criteria, articles had to be written in English and had to examine cognitive or functional or neuropsychiatric symptoms in relation to comorbidity in individuals diagnosed with LOAD (age 65 or over at onset)."*	4
Information sources	7	Describe all information sources (e.g., databases with dates of coverage, contact with study authors to identify additional studies) in the search and date last searched.<br>*"The articles were identified using the electronic databases Medline, EMBASE, PsycINFO and Cochrane updated until January 2016. (…)No restriction for years of publication was used."*	4

S6 Appendix. PRISMA Checklist.

Search	8	Present full electronic search strategy for at least one database, including any limits used, such that it could be repeated.	Appendix S1-S4
Study selection	9	State the process for selecting studies (i.e., screening, eligibility, included in systematic review, and, if applicable, included in the meta-analysis).<br>*“The title and abstract of the 3061 articles were independently screened by two reviewers (L.V., M.H.)”*	4
Data collection process	10	Describe method of data extraction from reports (e.g., piloted forms, independently, in duplicate) and any processes for obtaining and confirming data from investigators.<br>*“full text assessment which was performed in duplicate as well (L.V., M.H.).”*	4
Data items	11	List and define all variables for which data were sought (e.g., PICOS, funding sources) and any assumptions and simplifications made.<br>*“The keywords “Alzheimer” and “observational studies” and “progression” and “comorbidity” were used in subsequent combinations with either “cognition” or “daily functioning” or “behavior disorders”, along with their synonyms.”*	4
Risk of bias in individual studies	12	Describe methods used for assessing risk of bias of individual studies (including specification of whether this was done at the study or outcome level), and how this information is to be used in any data synthesis.<br>*“using the Newcastle-Ottawa quality assessment for cohort studies which assesses the selection of subjects, methods to control for confounding and assessment of the outcome.”*	4
Summary measures	13	State the principal summary measures (e.g., risk ratio, difference in means).<br>*No meta-analysis was performed*	NA
Synthesis of results	14	Describe the methods of handling data and combining results of studies, if done, including measures of consistency (e.g., I^2) for each meta-analysis.<br>*No meta-analysis was performed*	NA

Section/topic	#	Checklist item	Reported on page #
Risk of bias across studies	15	Specify any assessment of risk of bias that may affect the cumulative evidence (e.g., publication bias, selective reporting within studies).<br>*“During the critical appraisal of the studies, some methodological challenges emerged….”*	13-14
Additional analyses	16	Describe methods of additional analyses (e.g., sensitivity or subgroup analyses, meta-regression), if done, indicating which were pre-specified.<br>*No meta-analysis was performed*	NA
**RESULTS**			

S6 Appendix. PRISMA Checklist.

Study selection	17	Give numbers of studies screened, assessed for eligibility, and included in the review, with reasons for exclusions at each stage, ideally with a flow diagram.	4 (Fig.1)
Study characteristics	18	For each study, present characteristics for which data were extracted (e.g., study size, PICOS, follow-up period) and provide the citations.	6-9
Risk of bias within studies	19	Present data on risk of bias of each study and, if available, any outcome level assessment (see item 12).<br>*"After quality assessment, only one study was deemed to be of low quality with a score below 70%...."*	10-11<br>Appendix S5
Results of individual studies	20	For all outcomes considered (benefits or harms), present, for each study: (a) simple summary data for each intervention group (b) effect estimates and confidence intervals, ideally with a forest plot.	7-9
Synthesis of results	21	Present results of each meta-analysis done, including confidence intervals and measures of consistency.<br>*No meta-analysis was performed*	NA
Risk of bias across studies	22	Present results of any assessment of risk of bias across studies (see Item 15).<br>*"During the critical appraisal of the studies, some methodological challenges emerged…."*	13-14
Additional analysis	23	Give results of additional analyses, if done (e.g., sensitivity or subgroup analyses, meta-regression [see Item 16]).<br>*No meta-analysis was performed*	NA
**DISCUSSION**			
Summary of evidence	24	Summarize the main findings including the strength of evidence for each main outcome; consider their relevance to key groups (e.g., healthcare providers, users, and policy makers).<br>*"Although the total evidence available for this review was limited, the main finding was that increased somatic comorbid disease burden was associated with increased cognitive, functional and neuropsychiatric symptoms in LOAD."*	10
Limitations	25	Discuss limitations at study and outcome level (e.g., risk of bias), and at review-level (e.g., incomplete retrieval of identified research, reporting bias).	13-15
Conclusions	26	Provide a general interpretation of the results in the context of other evidence, and implications for future research.	15-16
**FUNDING**			
Funding	27	Describe sources of funding for the systematic review and other support (e.g., supply of data); role of funders for the systematic review.	uploaded

From: Moher D, Liberati A, Tetzlaff J, Altman DG, The PRISMA Group (2009). Preferred Reporting Items for Systematic Reviews and Meta-Analyses: The PRISMA Statement. PLoS Med 6(7): e1000097. doi:10.1371/journal.pmed1000097

For more information, visit: **www.prisma-statement.org**.
