# Supplementary material for: Comorbidity and progression of late onset Alzheimer’s disease: A systematic review
Source: PLoS One. 2017 May 4;12(5):e0177044. doi: 10.1371/journal.pone.0177044 (PMC5417646; doi:10.1371/journal.pone.0177044)
Supplement: S7 Appendix — (PDF) [file pone.0177044.s007.pdf]

S7 Appendix. Review Protocol.

Table of contents

1. Introduction and study objectives……………………………………………………………………...3
2. Eligibility criteria……………………………………………………………………………………………….6
3. Information sources…………………………………………………………………………………………7
4. Search……..8
5. Study selection………………………………………………………………………………………………..13
6. Data extraction……………………………………………………………………………………………….14
7. Data synthesis and analysis………………………………………………………………………….15
8. Reporting results…………………………………………………………………………………………..16
9. Appendix 1…………………………………………………………………………………………………….19
10. Appendix 2…………………………………………………………………………………………………..24
11. Appendix 3…………………………………………………………………………………………………..30
12. Appendix 4…………………………………………………………………………………………………..34
13. References…………………………………………………………………………………………………..35

1. Introduction and study objectives

Introduction

Dementia is typically defined as a clinical syndrome of cognitive decline that is sufficiently severe to interfere with social or occupational functioning[1]. It is an umbrella term for a group of cognitive disorders characterized by progressive decline in cognitive function due to brain damage or disease http://www.cdc.gov/mentalhealth/basics/mental-illness/dementia.htm. But above all, it has become an epidemic as its prevalence is increasing rapidly. The WHO-AD International report estimated that 35.56 million people suffered from dementia in 2010. This number is estimated to nearly double every 20 years, to 65.7 million in 2030, and 115.4 million in 2050 [2]. Alzheimer's disease (AD) continues to be the commonest cause of dementia in late life and accounts for 50-70% of the cases[3, 4]. AD is a neurodegenerative disease characterized by multidimensional progression which consists of a combination of cognitive, functional and psychiatric decline[5, 6]. It causes severe suffering, including deterioration in memory, thinking, behavior and the ability to perform everyday activities[1].

There is a substantial amount of variability in rates of decline among individuals with AD and knowing which are the influencing factors would be useful for understanding disease progression, as well as for resource planning and individual prognosis[7]. Potentially influencing factors are comorbidities. Comorbidity is defined as the coexistence of two or more chronic conditions and has increased with the decline in mortality rates combined with an aging population[8]. In the USA, the prevalence of comorbidity among Medicare (USA national social insurance program) recipients increases from 62% at age 65 to74 years to 82% at ages 85 years and older[9].

Comorbidities seem to increase dementia severity[10]. Several vascular related factors and conditions (i.e., hypertension, hypercholesterolemia, atherosclerosis, myocardial infarction, atrial fibrillation, diabetes) have been linked to increased risk of dementia and AD[11]. Besides, clinical experience suggests that dementia patients with physical comorbidities progress faster than patients without comorbidities [12]. This relation may offer a 'window' for slowing down dementia progression. Clinicians should be able to provide the patient and their family with a reliable prediction of the course of the disease but this is currently impossible, as we are facing a heterogeneous syndrome that progresses differently in each patient[13]. Many studies [14-17] report comorbidities effects on either cognitive, functional or neuropsychiatric measures in dementia patients, however AD must not be seen as a unidimensional disease. Consequently, predictions should take into account cognitive as well as functional and psychiatric aspects. Therefore the purpose of this review is to investigate which comorbidities affect the multidimensional progression rate in elderly patients with AD.

Research question
In diagnosed Alzheimer`s patients over 65 years old, which comorbidities predict the multidimensional progression of dementia?

D diagnosed Alzheimer`s patients (age > 65)
D comorbidity/multimorbidity
O multidimensional progression of dementia (cognition, functioning, psychiatric)

Dementia: is an umbrella term for a group of cognitive disorders typically characterized by progressive decline in cognitive function due to brain damage or disease. It is typically defined as a clinical syndrome of cognitive decline that is sufficiently severe to interfere with social or occupational functioning. Routine clinical practice shows that the cognitive and functional changes of dementia are typically accompanied by changes in behavior and in personality, which defines it as a multidimensional syndrome. [1]
Alzheimer's disease: Neurodegenerative disease (progressive nervous system dysfunction and loss of neural tissue) characterized by the progressive decline in cognition, functioning and psychiatric health. It's a syndrome in which there is deterioration in memory, thinking, behavior and the ability to perform everyday activities. [1]
Elderly: all patients age 65 or over.
Comorbidity/ Multimorbidity: Coexistence of two or more physical and/or mental disorders. Two or more medical conditions existing simultaneously and interdependently with each other, what means that one medical condition causes, is caused, or is otherwise related to another condition in the same individual; presence of additional diseases in relation to an index disease; presence of two or more diseases in some individual which are associated with each other through pathogenetic mechanisms and more frequently than it would be expected by chance (the inevitable side).[18]
Cognition: Act or process of knowing, which includes awareness and judgment, perceiving, reasoning, and conceiving; the mental process by which external or internal input is transformed, reduced, elaborated, stored, recovered, and used. As such, it involves a variety of functions such as perception, attention, memory coding, retention, and recall, decision-making, reasoning, problem-solving, imaging, planning and executing actions.[19]
Functioning/Functional status/Ability level/Independence: Demonstrated level of performance. Used in academic, cognitive, perceptual, or occupational contexts, as well as an indicator of a patient's level of functioning; individual's ability to carry out activities of daily living (ADLs) and to participate in various life situations and in society. Defined this way, it includes one's abilities in physical, emotional, and cognitive dimensions. Functional assessment, then, refers to the process of identifying and describing where on a continuum an individual's functional status lies.[20]
Activities of Daily Living (ADLs)/Self Care Skills: Personal care skills used to measure functional ability in the elderly and the emotionally and physically disabled. Are a defined set of activities necessary for normal self-care can be classified into basic activities of daily living (BADL) and instrumental activities of daily living (IADL). **BADL** are composed of more basic, self-care behaviors such as ambulating, eating, dressing, grooming, bathing, feeding, and toileting *(we can see ADL and BADL used as synonyms in many articles).* **IADLs** are activities related to independent living and involve interaction with the physical and social environment, generally more complex than personal ADL. IADLs facilitate independent living through behaviors such as transportation, telephone use, meal preparation, medication management, financial management, housekeeping, laundry, and shopping.[20]

Neuropsychiatric Symptoms (NPS): Psychiatric symptoms caused by organic brain disorders could be called "neuropsychiatric" symptoms. NPS can be defined as psychiatric manifestations of cerebral (neuropsychiatric) disorders. NPS/syndromes are also classified as follows: cognitive disorders (dementias and predementia syndromes, nondementing cognitive disorders), seizure disorders, movement disorders, traumatic brain injury, secondary psychiatric disorders (psychosis, depression, mania and anxiety disorders secondary to "organic" brain disease), substance-induced psychiatric disorders, attention disorders, and sleep disorders. **NPS-AD** are highly prevalent (80% to 90%), and most individuals with AD will exhibit one or multiple symptoms over the course of the illness. Symptoms can range from mild (depression, anxiety, irritability, and apathy) to severe (agitation, aggression, aberrant vocalizations, hallucinations, and disinhibition, among others).[21]

2. Eligibility criteria

Criteria	Inclusion	Exclusion
Domain	• Age >65<br>• Alzheimer's disease patients (already diagnosed)	• Age < 65<br>• Other dementia patients<br>• Non diagnosed Alzheimer's patients
Determinants	• Predictors of decline<br>• Comorbidity/Multimorbidity<br>• Other diseases than AD which predict AD progression	• Predictors of dementia incidence
Outcomes	Multidimensional progression:<br>• Cognitive decline<br>• Functional (ADLs) decline<br>• Psychiatric decline	• Survival<br>• Institutionalization<br>• Treatment effect<br>• Quality of life
Setting		
Study design	• All types of prospective cohort studies	• Retrospective studies<br>• Clinical trials<br>• Case reports<br>• Opinions<br>• Commentaries<br>• Letters to the editor with no primary data
Language	• English language	• Other than English language
Year of publication	• All	• None

3. Information sources

Studies will be identified using the electronic databases Medline, EMBASE, PsycINFO and Cochrane up to January 2016. For Medline a comprehensive search strategy is developed (see section 4), which will be adapted for PsycINFO, EMBASE and Cochrane (see appendix 1, 2 and 3). The snowball method will be used to manually identify relevant references from the reference lists of included articles.

4. Search

The research strategy will include relevant synonyms for:
1) Alzheimer's Disease
2) Comorbidities
3) Multidimensional progression (cognition, functioning, neuropsychiatric symptoms)

MEDLINE TOOLS:

- kf: keyword heading word
- kw: keyword heading
- ti: title
- ab: abstract
- tw (ti+ab): text word

1) Alzheimer's Disease

1. exp Alzheimer Disease/ or alzheimer*.sh.
2. (alzheimer sclerosis or alzheimer disease late onset or alzheimer type dementia or dementia primary senile degenerative or presenile alzheimer dementia or alzheimer syndrome or dementia alzheimer-type or senile dementia or dementia alzheimer or dementia alzheimer type or presenile dementia or dementia senile or late onset alzheimer disease or alzheimer's disease or primary senile degenerative dementia or syndrome alzheimer or dementia presenile or alzheimer disease assessment scale or alzheimer's disease or alzheimer's-disease or alzheimer-disease).tw.
3. (alzheimer sclerosis or alzheimer disease late onset or alzheimer type dementia or dementia primary senile degenerative or presenile alzheimer dementia or alzheimer syndrome or dementia alzheimer-type or senile dementia or dementia alzheimer or dementia alzheimer type or presenile dementia or dementia senile or late onset alzheimer disease or alzheimer's disease or primary senile degenerative dementia or syndrome alzheimer or dementia presenile or alzheimer disease assessment scale or alzheimer's disease or alzheimer's-disease or alzheimer-disease).kf.
4. exp Dementia/ or dementia.sh.
5. (amentia* or dement* senile paranoid or dementia presenile or mental deterioration).tw.
6. (amentia* or dement* senile paranoid or dementia presenile or mental deterioration).kf.
7. 1 or 2 or 3 or 4 or 5 or 6

2) Observational/Prognosis/Predictor/Comorbidity

8. "Observational studies".ti. or epidemiologic studies/ or exp case-control studies/ or cross-sectional studies/
9. ((case adj3 control) or (cohort adj5 (study or studies or analy$)) or (follow-up adj5 (study or studies)) or (longitudinal or retrospective or prospective or (cross adj5 sectional)) or (observational adj5 (study or studies))).af.
10. 8 or 9
11. 7 and 10
12. exp Prognosis/ or prognos*.sh.
13. exp Probability/ or probab*.sh.
14. exp Proportional Hazards Models/

15. exp Likelihood Functions/
16. exp Logistic Models/
17. exp Forecasting/ or forecast*.sh.
18. exp Risk Factors/ or risk factor*.sh.
19. (decision support techniques or prediction or predictive validity or predictor variable).tw.
20. (decision support techniques or prediction or predictive validity or predictor variable).kf.
21. ((risk adj prediction) or (predictor adj variabl??) or (increas* adj risk)).mp. or ((risk adj assessement?) or (predict* adj risk?) or (risk adj factor?) or (validat* or predict* or rule*)).tw.
22. ((risk adj prediction) or (predictor adj variabl??) or (increas* adj risk)).mp. or ((risk adj assessement?) or (predict* adj risk?) or (risk adj factor?) or (validat* or predict* or rule*)).kf.
23. ((predict* and (outcome* or risk* or model*)) or ((history or variable* or criteria or scor* or characteristic* or finding* or factor*) and (predict* or model* or decision* or identi* or prognos*)) or (decision* and (model* or clinical* or (logistic adj3 models)))).tw. or ((predict* and (outcome* or risk* or model*)) or ((history or variable* or criteria or scor* or characteristic* or finding* or factor*) and (predict* or model* or decision* or identi* or prognos*)) or (decision* and (model* or clinical* or (logistic adj3 models)))).kf.
24. 12 or 13 or 14 or 15 or 16 or 17 or 18 or 19 or 20 or 21 or 22 or 23
25. exp Comorbidity/ or comorbidit*.sh.
26. (multimorbidit* or comorbidit*).tw.
27. (multimorbidit* or comorbidit*).kf.
28. (co morbidit* or co-morbidit* or coomorbidit*).tw.
29. (co morbidit* or co-morbidit* or coomorbidit*).kf.
30. (chronic diseases or cooccurring diseases or co-occurring diseases or co occurring diseases or clusters of diseases or disease burden or physical health or medical health or charlson or cumulative illness scale geriatrics or polymorbidity or disease count).tw.
31. (chronic diseases or cooccurring diseases or co-occurring diseases or co occurring diseases or clusters of diseases or disease burden or physical health or medical health or charlson or cumulative illness scale geriatrics or polymorbidity or disease count).kf.
32. 25 or 26 or 27 or 28 or 29 or 30 or 31
33. 24 and 32

3) Multidimensional progression

34. exp Cognition/ or cognit*.sh.
35. exp Cognition Disorders/ or cognit* disorder*.sh.
36. cognit*.tw.
37. cognit*.kf.
38. (consciousness disorders or overinclusion or cognitive performance or confusion or intellectual disability or perceptual disorders or mental competency or perception or thinking or aptitude or mild cognitive impairment or cognitive defect or cognitive generalization or cognitive complexity or cognitive contiguity or cognitive dissonance or cognitive appraisal or cognitive maps or wayfinding or spatial imagery or direction perception or thought content or cognitive functioning or executive functioning or intellectual functioning or mathematical ability or reading ability or verbal ability or cognitive deficits or cognitive dysfunction or executive dysfunction or thought disturbances or human information process or cognitive science or information processing model or metacognition or intelligence or intelligence measures or intelligence quotient).tw. or (consciousness disorders or overinclusion or cognitive performance or confusion or intellectual disability or perceptual disorders or mental competency or perception or thinking or aptitude or mild cognitive impairment or cognitive defect or cognitive generalization or cognitive complexity or cognitive contiguity or

cognitive dissonance or cognitive appraisal or cognitive maps or wayfinding or spatial imagery or direction perception or thought content or cognitive functioning or executive functioning or intellectual functioning or mathematical ability or reading ability or verbal ability or cognitive deficits or cognitive dysfunction or executive dysfunction or thought disturbances or human information process or cognitive science or information processing model or metacognition or intelligence or intelligence measures or intelligence quotient).kf.
39. exp Spatial Memory/ or exp Memory/ or exp Memory, Long-Term/ or exp Memory, Episodic/ or exp Memory Disorders/ or exp Memory, Short-Term/ or (spatial memory or memory or memory, long-term or memory, episodic or memory disorder* or memory, short-term).sh.
40. (spatial memory disorders or memory disorder or memory losses or memory disorders age related or retention disorder cognitive or memory disorder semantic or age-related memory disorder or memory deficits or spatial memory disorder or cognitive retention disorder or memories autobiographical or memories episodic or memories prospective or longterm memories or memories remote or memory long-term or immediate memories or short-term memory or working memory or memory shortterm or recall immediate or verbal memory or visual memory or amnesia or anterograde amnesia or global amnesia or retrograde amnesia or memory consolidation or memory decay or memory trace or visuospatial memory or associative memory or auditory memory or false memory or olfactory memory or recognition or reference memory or repetition priming or retrospective memory or sensory memory or tactile memory or memory bias or word list recall or word recognition or working memory).tw. or (spatial memory disorders or memory disorder or memory losses or memory disorders age related or retention disorder cognitive or memory disorder semantic or age-related memory disorder or memory deficits or spatial memory disorder or cognitive retention disorder or memories autobiographical or memories episodic or memories prospective or longterm memories or memories remote or memory long-term or immediate memories or short-term memory or working memory or memory shortterm or recall immediate or verbal memory or visual memory or amnesia or anterograde amnesia or global amnesia or retrograde amnesia or memory consolidation or memory decay or memory trace or visuospatial memory or associative memory or auditory memory or false memory or olfactory memory or recognition or reference memory or repetition priming or retrospective memory or sensory memory or tactile memory or memory bias or word list recall or word recognition or working memory).kf.
41. exp Language/ or exp Language Disorders/ or exp Speech-Language Pathology/ or (language or language disorders or speech-language pathology).sh.
42. exp Verbal Behavior/ or verbal behavior.sh.
43. (language disorder acquired or pathology speech or pathology language or verbal fluency or "speech and language" or language or linguistic or "speech and language assessment" or language ability or oral communication or speech analysis or speech perception or speech disorder or language disability or language ability or language test or communication disorders).tw. or (language disorder acquired or pathology speech or pathology language or verbal fluency or "speech and language" or language or linguistic or "speech and language assessment" or language ability or oral communication or speech analysis or speech perception or speech disorder or language disability or language ability or language test or communication disorders).kf.
44. exp Executive Function/ or executive function*.sh.
45. exp Attention/ or attention.sh.
46. exp Problem Solving/ or problem solving.sh.
47. exp Decision Making/ or decision making.sh.
48. exp Orientation/ or orientation.sh.
49. exp Reading/ or reading.sh.
50. exp Judgment/ or judgment.sh.

51. (executive control or concentration or shared decision making or mental speed or verbal reasoning or abstraction).tw. or (executive control or concentration or shared decision making or mental speed or verbal reasoning or abstraction).kf.
52. 34 or 35 or 36 or 37 or 38 or 39 or 40 or 41 or 42 or 43 or 44 or 45 or 46 or 47 or 48 or 49 or 50 or 51

53. exp Motor Activity/ or exp "Activities of Daily Living"/ or activit* of daily living.sh. or motor activit*.sh.
54. exp Self Care/ or self care.sh.
55. exp Walking/ or walking.sh.
56. exp Locomotion/ or locomot*.sh.
57. exp Postural Balance/ or postural balance*.sh.
58. exp Hand Strength/ or hand strength.sh.
59. exp Physical Endurance/ or physical endurance.sh.
60. exp Motor Activity/
61. exp Motor Skills/ or motor skill*.sh.
62. exp Gait/ or exp Gait Disorders, Neurologic/ or (gait or gait disorder*).sh.
63. exp Compressive Strength/ or exp Muscle Strength/ or (compressive strength or muscle strength).sh.
64. exp Accidental Falls/ or accidental fall*.sh.
65. exp Personal Autonomy/ or personal autonomy.sh.
66. exp Work Capacity Evaluation/ or work capacity evaluation.sh.
67. (physical function or ambulation or limitation of activity chronic or self management or self-care or self-management or musculoskeletal equilibrium or postural equilibrium or grasps or grips or pinch strength or (physical function or ambulation or limitation of activity chronic or self management or self-care or self-management or musculoskeletal equilibrium or postural equilibrium or grasps or grips or pinch strength) or (physical function or ambulation or limitation of activity chronic or self management or self-care or self-management or musculoskeletal equilibrium or postural equilibrium or grasps or grips or pinch strength)).tw. or (physical function or ambulation or limitation of activity chronic or self management or self-care or self-management or musculoskeletal equilibrium or postural equilibrium or grasps or grips or pinch strength).kf.
68. 53 or 54 or 55 or 56 or 57 or 58 or 59 or 60 or 61 or 62 or 63 or 64 or 65 or 66 or 67

69. exp Mental Disorders/ or mental disorder*.sh.
70. exp Geriatric Psychiatry/ or *Psychiatry/ or geriatr* psychiat*.sh. or psychiatr*.sh.
71. exp Behavioral Symptoms/ or behavior symptom*.sh.
72. exp Anxiety/ or exp Anxiety Disorders/ or (anxiety or anxiety disorder*).sh.
73. confusion/ or exp delirium/ or (confusion or delirium).sh.
74. emotions/ or affect/ or anger/ or apathy/ or hostility/ or (emotions or affect or anger or apathy or hostility).sh.
75. exp Delirium, Dementia, Amnestic, Cognitive Disorders/ or exp Psychotic Disorders/ or (delirium, dementia, amnestic, cognitive disorders or psychotic disorders).sh.
76. exp Aggression/ or aggress*.sh.
77. exp Hostility/
78. exp Behavior Control/ or exp Illness Behavior/ or exp Social Behavior Disorders/ or (behavior control or illness behavior or social behavior disorders).sh.
79. exp Delusions/ or delusion*.sh.
80. exp Hallucinations/ or hallucination*.sh.

81. exp Psychomotor Agitation/ or psychomotor agitation.sh.
82. exp Depression/ or depression.sh.
83. exp Euphoria/ or euphoria.sh.
84. exp Apathy/ or apathy.sh.
85. exp Irritable Mood/ or irritable mood.sh.
86. (emotional disorder or mental patient or mental instability or mood disorder or organic psychosyndrome or dysphoria or neuropsychological assessment or psychiatric evaluation or psychiatric disorders or indifference or disinhibition or lability or aberrant motor behavior or hallucinat* or agitat* or anxiety elation or apath* or neuropsychiat*).tw. or (emotional disorder or mental patient or mental instability or mood disorder or organic psychosyndrome or dysphoria or neuropsychological assessment or psychiatric evaluation or psychiatric disorders or indifference or disinhibition or lability or aberrant motor behavior or hallucinat* or agitat* or anxiety elation or apath* or neuropsychiat*).kf.
87. 69 or 70 or 71 or 72 or 73 or 74 or 75 or 76 or 77 or 78 or 79 or 80 or 81 or 82 or 83 or 84 or 85 or 86

88. exp Disease Progression/ or disease progress*.sh.
89. exp Patient Outcome Assessment/ or exp Fatal Outcome/ or exp "Outcome Assessment (Health Care)"/ or exp "Outcome and Process Assessment (Health Care)"/ or ((patient outcome assessment or fatal outcome or outcome assessment or outcome) and process assessment).sh.
90. exp Chronic Disease/ or chronic disease.sh.
91. exp Survival Analysis/ or *Survival/ or exp Survival Rate/ or (survival analysis or *survival/ or survival rate).sh.
92. exp "Age of Onset"/ or age of onset.sh.
93. exp Terminal Care/ or terminal care.sh.
94. (disease control or disease course or adverse outcome or chronicity or deterioration or disease duration or disease exacerbation or general condition deterioration or general condition improvement or illness trajectory or remission or progress* or impairment or decline or failure or decrease or worsening or deterioration or degeneration).tw. or (disease control or disease course or adverse outcome or chronicity or deterioration or disease duration or disease exacerbation or general condition deterioration or general condition improvement or illness trajectory or remission or progress* or impairment or decline or failure or decrease or worsening or deterioration or degeneration).kf.
95. 88 or 89 or 90 or 91 or 92 or 93 or 94

Final combinations:

96. 11 and 32 and 95
97. 52 and 96
98. 68 and 96
99. 87 and 96

5. Study selection

The results of the search strategy will be screened for duplicates by one researcher (LRV). These duplicates will be removed. Then, the researcher (LRV) will pursue the screening by independently examining each title and available abstract. Subsequently, studies that seem to meet the eligibility criteria or studies from which, based on the title/abstract, there is uncertainty about the eligibility, will be screened in full-text. This will be done independently by two researchers (LRV and MH). The **snowball method** will be used in the included studies to identify relevant references from the reference list. When there is uncertainty about the method or additional information is required, the (corresponding) authors of the articles will be approached. If necessary, the hand search method will also be used.

6. Data extraction

Data collection includes information about: methods, domain, determinants, outcomes measures and results.

Quality assessment

The studies will go through a stage of quality assessment using pre defined criteria.
The articles will be assessed with an emphasis on validity and reliability.
Risk of bias: the Critical Appraisal Prognostic Studies will be used on all selected articles (see Appendix 4). Quality assessment tools will be completed by two researchers independently (LRV and MH). Outcomes will be compared, and in case of differences will be discussed. If needed, a third researcher (RM) will be consulted to obtain consensus.

7. Data synthesis and analysis

It will not be feasible to conduct a meta-analysis, due to the expected heterogenic determinants, subject groups and outcome measures. Instead we will give a narrative overview of our findings. Results will also be reported in tables.

8. Reporting results

Study results will be reported according to the Prisma statement (see Figure 1).

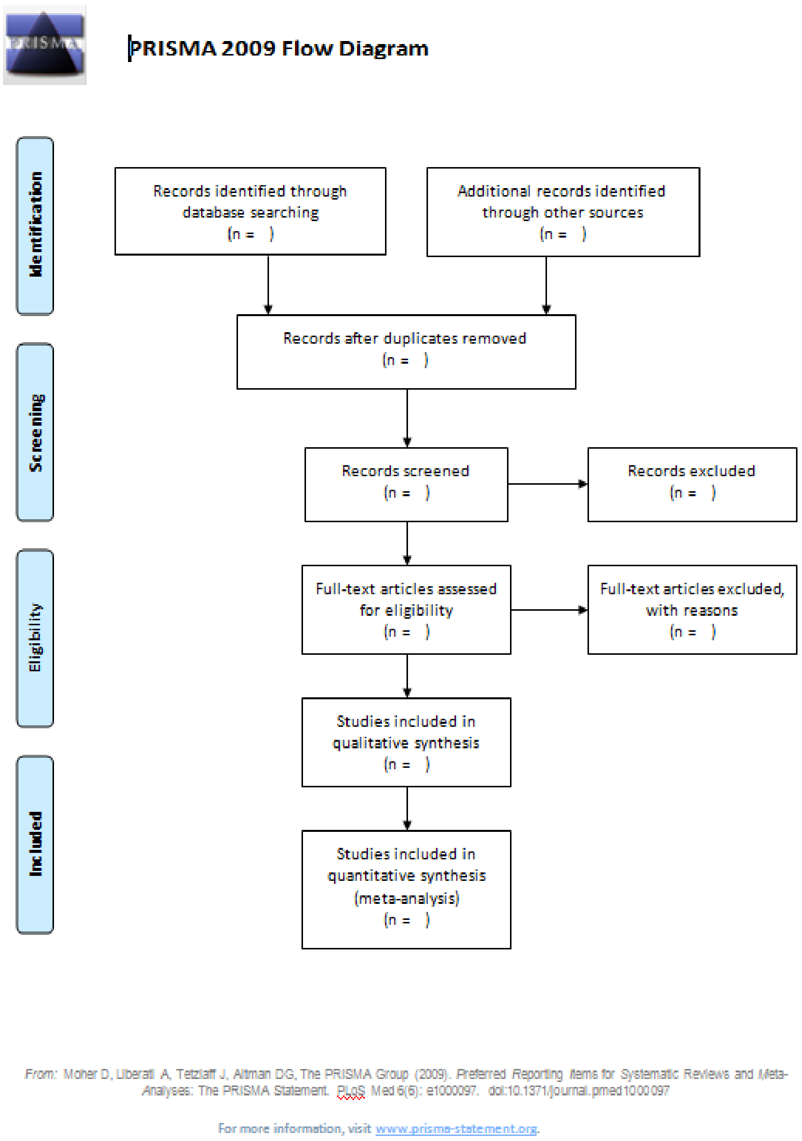

Figure 1. PRISMA Flow diagram

The study characteristics will be presented in a table. Also, relevant study characteristics (such as study size, age and comorbidities) will be addressed in a narrative summary of the studies.
An overview of the risk bias of all included studies will be provided in a bias graph (see Figure 2).

Example of a risk of bias graph

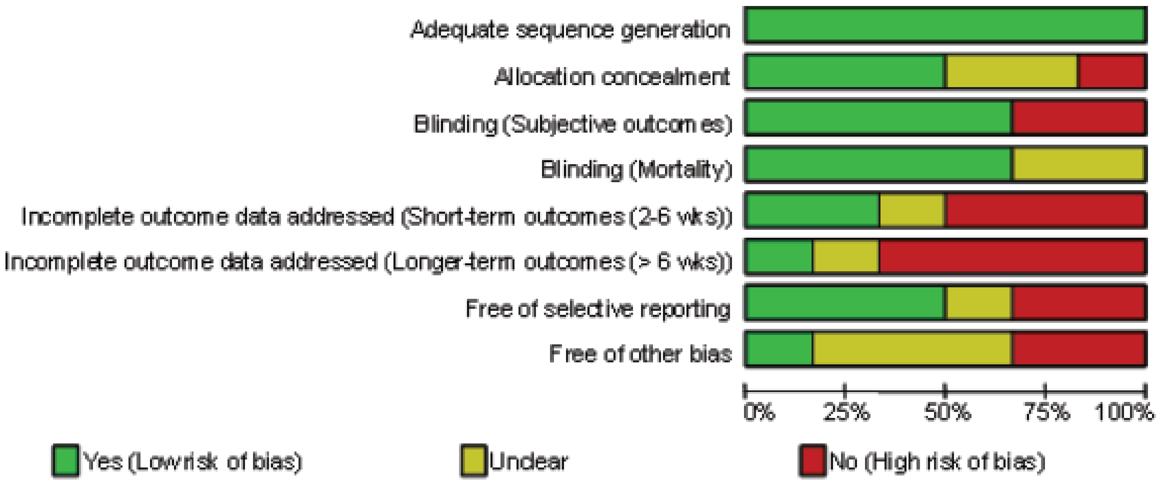

Figure 2. Risk of bias graph

9. Appendix 1
Search PsycINFO

PsycINFO TOOLS:
- id: key concepts
- ti: title
- ab: abstract
- sh: subject heading
- tw (ti+ab): text word

1) Alzheimer's Disease

1. exp Alzheimer's Disease/ or alzheimer*.sh.
2. (alzheimer sclerosis or alzheimer disease late onset or alzheimer type dementia or dementia primary senile degenerative or presenile alzheimer dementia or alzheimer syndrome or dementia alzheimer-type or senile dementia or dementia alzheimer or dementia alzheimer type or presenile dementia or dementia senile or late onset alzheimer disease or alzheimer's disease or primary senile degenerative dementia or syndrome alzheimer or dementia presenile or alzheimer disease assessment scale or alzheimer's disease or alzheimer's-disease or alzheimer-disease).tw. or (alzheimer sclerosis or alzheimer disease late onset or alzheimer type dementia or dementia primary senile degenerative or presenile alzheimer dementia or alzheimer syndrome or dementia alzheimer-type or senile dementia or dementia alzheimer or dementia alzheimer type or presenile dementia or dementia senile or late onset alzheimer disease or alzheimer's disease or primary senile degenerative dementia or syndrome alzheimer or dementia presenile or alzheimer disease assessment scale or alzheimer's disease or alzheimer's-disease or alzheimer-disease).id.
3. exp Dementia/ or dementia.sh.
4. (amentia* or dement* senile paranoid or dementia presenile or mental deterioration).tw. or (amentia* or dement* senile paranoid or dementia presenile or mental deterioration).id.
5. 1 or 2 or 3 or 4

2) Observational/Prognosis/Predictor/Comorbidity

6. Observational studies.ti. or epidemiologic studies/ or exp case-control studies/ or cross-sectional studies/
7. ((case adj3 control) or (cohort adj5 (study or studies or analy$)) or (follow-up adj5 (study or studies)) or (longitudinal or retrospective or prospective or (cross adj5 sectional)) or (observational adj5 (study or studies))).af.
8. 6 or 7
9. 5 and 8
10. exp Prognosis/ or prognos*.sh.
11. exp Probability/ or probab*.sh.
12. exp Prediction/ or predict*.sh.
13. exp Risk Factors/ or risk factor*.sh.
14. (decision support techniques or forecasting or predictive validity or predictor variable).tw. or (decision support techniques or forecasting or predictive validity or predictor variable).id.
15. ((risk adj prediction) or (predictor adj variabl??) or (increas* adj risk)).mp. or ((risk adj assessement?) or (predict* adj risk?) or (risk adj factor?) or (validat* or predict* or rule*)).tw. or (((risk adj prediction) or (predictor adj variabl??) or (increas* adj risk)).mp. or ((risk adj assessement?) or (predict* adj risk?) or (risk adj factor?) or (validat* or predict* or rule*)).id.)

16. ((predict* and (outcome* or risk* or model*)) or ((history or variable* or criteria or scor* or characteristic* or finding* or factor*) and (predict* or model* or decision* or identi* or prognos*)) or (decision* and (model* or clinical* or (logistic adj3 models)))).tw. or ((predict* and (outcome* or risk* or model*)) or ((history or variable* or criteria or scor* or characteristic* or finding* or factor*) and (predict* or model* or decision* or identi* or prognos*)) or (decision* and (model* or clinical* or (logistic adj3 models)))).id.
17. 10 or 11 or 12 or 13 or 14 or 15 or 16
18. exp Comorbidity/ or comorbidit*.sh.
19. (multimorbidit* or comorbidit*).tw. or (multimorbidit* or comorbidit*).id.
20. (co morbidit* or co-morbidit* or coomorbidit*).tw. or (co morbidit* or co-morbidit* or coomorbidit*).id.
21. (chronic diseases or cooccurring diseases or co-occurring diseases or co occurring diseases or clusters of diseases or disease burden or physical health or medical health or charlson or cumulative illness scale geriatrics or polymorbidity or disease count).tw. or (chronic diseases or cooccurring diseases or co-occurring diseases or co occurring diseases or clusters of diseases or disease burden or physical health or medical health or charlson or cumulative illness scale geriatrics or polymorbidity or disease count).id.
22. 18 or 19 or 20 or 21
23. 17 and 22

3) Multidimensional progression

24. exp Cognition/ or cognit*.sh.
25. exp Cognitions/
26. exp cognitive ability/ or cognitive ability.sh.
27. exp spatial ability/ or spatial ability.sh.
28. exp cognitive appraisal/ or cognitive appraisal.sh.
29. exp cognitive assessment/ or cognitive assessment.sh.
30. exp cognitive control/ or cognitive control.sh.
31. exp Cognitive Dissonance/ or cognitive dissonance.sh.
32. exp Cognitive Impairment/ or cognitive impairment.sh.
33. exp cognitive maps/ or wayfinding/ or direction perception/ or mental models/ or schema/ or spatial imagery/ or "spatial orientation (perception)"/ or (cognitive maps or wayfinding or direction perception or mental models or schema or spatial imagery or spatial orientation).sh.
34. exp cognitive processes/ or cognitive processes.sh.
35. cognit*.tw. or cognit*.id.
36. (consciousness disorders or overinclusion or cognitive performance or confusion or intellectual disability or perceptual disorders or mental competency or perception or thinking or aptitude or mild cognitive impairment or cognitive defect or cognitive generalization or cognitive complexity or cognitive contiguity or thought content or cognitive functioning or executive functioning or intellectual functioning or mathematical ability or reading ability or verbal ability or cognitive deficits or cognitive dysfunction or executive dysfunction or thought disturbances or human information process or cognitive science or information processing model or metacognition or intelligence or intelligence measures or intelligence quotient or expectations or irrational beliefs).tw. or (consciousness disorders or overinclusion or cognitive performance or confusion or intellectual disability or perceptual disorders or mental competency or perception or thinking or aptitude or mild cognitive impairment or cognitive defect or cognitive generalization or cognitive complexity or cognitive contiguity or thought content or cognitive functioning or executive functioning or intellectual functioning or mathematical ability or reading ability or verbal ability or cognitive deficits

or cognitive dysfunction or executive dysfunction or thought disturbances or human information process or cognitive science or information processing model or metacognition or intelligence or intelligence measures or intelligence quotient or expectations or irrational beliefs).id.
37. exp Long Term Memory/ or exp Visuospatial Memory/ or exp False Memory/ or *Memory/ or exp Short Term Memory/ or exp Retrospective Memory/ or exp Visual Memory/ or exp Episodic Memory/ or exp Spatial Memory/ or exp Autobiographical Memory/ or exp Memory Disorders/ or exp Prospective Memory/ or exp Semantic Memory/ or exp Verbal Memory/ or (long term memory or visuospatial memory or false memory or *memory/ or short term memory or retrospective memory or visual memory or episodic memory or spatial memory or autobiographical memory or memory disorders or prospective memory or semantic memory or verbal memory).sh.
38. *Anterograde Amnesia/ or exp Amnesia/ or *Retrograde Amnesia/ or *Global Amnesia/ or (*anterograde amnesia/ or amnesia or *retrograde amnesia/ or *global amnesia/).sh.
39. exp memory decay/ or memory decay.sh.
40. exp memory trace/ or memory trace.sh.
41. (early memories or eidetic imagery or memory consolidation or reminiscence or "spontaneous recovery (learning)" or memory losses or memory disorders age related or retention disorder cognitive or memory disorder semantic or age-related memory disorder or remote memory or immediate memories or working memory or recall immediate or associative memory or auditory memory or olfactory memory or recognition or reference memory or repetition priming or sensory memory or tactile memory or memory bias or word list recall or word recognition).tw. or (early memories or eidetic imagery or memory consolidation or reminiscence or "spontaneous recovery (learning)" or memory losses or memory disorders age related or retention disorder cognitive or memory disorder semantic or age-related memory disorder or remote memory or immediate memories or working memory or recall immediate or associative memory or auditory memory or olfactory memory or recognition or reference memory or repetition priming or sensory memory or tactile memory or memory bias or word list recall or word recognition).id.
42. *Language/ or exp Speech Language Pathology/ or exp Language Disorders/ or (*language/ or speech language pathology or language disorders).sh.
43. exp Oral Communication/ or oral communication.sh.
44. exp Verbal Ability/ or exp Verbal Comprehension/ or exp Verbal Communication/ or (verbal ability or verbal comprehension or verbal communication).sh.
45. (speech-language pathology or verbal behavior or language disorder acquired or pathology speech or pathology language or verbal fluency or "speech and language" or linguistic or language ability or speech analysis or speech perception or speech disorder or language disability or language ability or language test or communication disorders or neurolinguistics).tw. or (speech-language pathology or verbal behavior or language disorder acquired or pathology speech or pathology language or verbal fluency or "speech and language" or linguistic or language ability or speech analysis or speech perception or speech disorder or language disability or language ability or language test or communication disorders or neurolinguistics).id.
46. exp Attention/ or attention.sh.
47. exp Problem Solving/ or problem solving.sh.
48. exp Decision Making/ or decision making.sh.
49. exp Reading/ or exp Reading Comprehension/ or exp Reading Ability/ or (reading or reading comprehension or reading ability).sh.
50. exp Perceptual Orientation/ or perceptual orientation.sh.
51. exp Judgment/ or exp Judgment Disturbances/ or (judgment or judgment disturbances).sh.
52. (executive function or executive control or concentration or shared decision making or mental speed or verbal reasoning or abstraction).tw. or (executive function or executive control or concentration or shared decision making or mental speed or verbal reasoning or abstraction).id.

53. 24 or 25 or 26 or 27 or 28 or 29 or 30 or 31 or 32 or 33 or 34 or 35 or 36 or 37 or 38 or 39 or 40 or 41 or 42 or 43 or 44 or 45 or 46 or 47 or 48 or 49 or 50 or 51 or 52

54. exp "activities of daily living"/ or activit* of daily living.sh.
55. exp Physical Activity/ or physical activit*.sh.
56. exp walking/ or exp motor performance/ or exp gait/ or (walking or motor performance or gait).sh.
57. exp Physical Strength/ or physical strength.sh.
58. exp Self Management/ or exp Self Care Skills/ or (self management or self care skills).sh.
59. exp Locomotion/ or locomot*.sh.
60. exp Equilibrium/ or exp Posture/ or exp Falls/ or exp Motor Coordination/ or exp Exercise/ or exp Motor Processes/ or exp Perceptual Motor Processes/ or (equilibrium or posture or falls or motor coordination or exercise or motor processes or perceptual motor processes).sh.
61. exp Physical Activity/ or exp Physical Disorders/ or exp Physical Endurance/ or exp Physical Health/ or exp Physical Mobility/ or exp Physical Agility/ or (physical activity or physical disorders or physical endurance or physical health or physical mobility or physical agility).sh.
62. exp Motor Performance/ or exp Motor Skills/ or (motor performance or motor skills).sh.
63. exp Physical Strength/ or physical strength.sh.
64. (hand strength or gait disorders or compressive strength or muscle strength or accidental falls or personal autonomy or work capacity evaluation or physical function or ambulation or limitation of activity chronic or self-care or self-management or musculoskeletal equilibrium or postural equilibrium or grasps or grips or pinch strength).tw. or (hand strength or gait disorders or compressive strength or muscle strength or accidental falls or personal autonomy or work capacity evaluation or physical function or ambulation or limitation of activity chronic or self-care or self-management or musculoskeletal equilibrium or postural equilibrium or grasps or grips or pinch strength).id.
65. 54 or 55 or 56 or 57 or 58 or 59 or 60 or 61 or 62 or 63 or 64

66. exp Mental Disorders/ or mental disorder*.sh.
67. exp Geriatric Psychiatry/ or *Psychiatry/ or geriatr* psychiat*.sh. or psychiatr*.sh.
68. exp Behavior Disorders/ or exp Behavior Modification/ or *Behavior/ or exp Behavior Problems/ or exp Behavior Change/ or exp Aggressive Behavior/ or exp Illness Behavior/ or (behavior disorders or behavior modification or *behavior/ or behavior problems or behavior change or aggressive behavior or illness behavior).sh.
69. exp Anxiety Disorders/ or exp Anxiety/ or (anxiety or anxiety disorder*).sh.
70. exp Delirium/ or exp Mental Confusion/ or (delirium or mental confusion).sh.
71. exp Emotions/ or emotion*.sh.
72. exp Emotional States/ or emotional state*.sh.
73. exp Aggressiveness/ or exp Aggressive Behavior/ or (aggressiveness or aggressive behavior).sh.
74. exp Hostility/ or hostility.sh.
75. exp Delusions/ or delusion*.sh.
76. exp Visual Hallucinations/ or exp Auditory Hallucinations/ or exp Hallucinations/ or (visual hallucinations or auditory hallucinations or hallucinations).sh.
77. exp Agitation/ or agitation.sh.
78. exp Major Depression/ or major depression.sh.
79. exp Euphoria/ or euphoria.sh.
80. exp Apathy/ or apathy.sh.
81. exp Irritability/ or irritability.sh.

82. (emotional disorder or mental patient or mental instability or mood disorder or organic psychosyndrome or dysphoria or neuropsychological assessment or psychiatric evaluation or psychiatric disorders or indifference or disinhibition or lability or aberrant motor behavior or hallucinat* or agitat* or anxiety elation or apath* or neuropsychiat*).tw. or (emotional disorder or mental patient or mental instability or mood disorder or organic psychosyndrome or dysphoria or neuropsychological assessment or psychiatric evaluation or psychiatric disorders or indifference or disinhibition or lability or aberrant motor behavior or hallucinat* or agitat* or anxiety elation or apath* or neuropsychiat*).id.
83. 66 or 67 or 68 or 69 or 70 or 71 or 72 or 73 or 74 or 75 or 76 or 77 or 78 or 79 or 80 or 81 or 82

84. exp Disease Course/ or disease course.sh.
85. exp Chronic Mental Illness/ or exp Chronic Illness/ or (chronic mental illness or chronic illness).sh.
86. exp "Onset (Disorders)"/ or onset disorder*.sh.
87. (disease progression or patient outcome assessment or fatal outcome or outcome assessment or process assessment or survival analysis or survival or survival rate or age of onset or terminal care or disease control or disease course or adverse outcome or chronicity or deterioration or disease duration or disease exacerbation or general condition deterioration or general condition improvement or illness trajectory or remission or progress* or impairment or decline or failure or decrease or worsening or deterioration or degeneration).tw. or (disease progression or patient outcome assessment or fatal outcome or outcome assessment or process assessment or survival analysis or survival or survival rate or age of onset or terminal care or disease control or disease course or adverse outcome or chronicity or deterioration or disease duration or disease exacerbation or general condition deterioration or general condition improvement or illness trajectory or remission or progress* or impairment or decline or failure or decrease or worsening or deterioration or degeneration).id.
88. 84 or 85 or 86 or 87

Final combinations:

89. 9 and 22 and 88
90. 53 and 89
91. 65 and 89
92. 83 and 89

10. Appendix 2
Search EMBASE

EMBASE TOOLS:
- kw: keyword
- ti: title
- ab: abstract
- tw (ti+ab): text word

1) Alzheimer's Disease

1. exp Alzheimer Disease/ or alzheimer*.sh.
2. (alzheimer sclerosis or alzheimer disease late onset or alzheimer type dementia or dementia primary senile degenerative or presenile alzheimer dementia or alzheimer syndrome or dementia alzheimer-type or senile dementia or dementia alzheimer or dementia alzheimer type or presenile dementia or dementia senile or late onset alzheimer disease or alzheimer's disease or primary senile degenerative dementia or syndrome alzheimer or dementia presenile or alzheimer disease assessment scale or alzheimer's disease or alzheimer's-disease or alzheimer-disease).tw. or (alzheimer sclerosis or alzheimer disease late onset or alzheimer type dementia or dementia primary senile degenerative or presenile alzheimer dementia or alzheimer syndrome or dementia alzheimer-type or senile dementia or dementia alzheimer or dementia alzheimer type or presenile dementia or dementia senile or late onset alzheimer disease or alzheimer's disease or primary senile degenerative dementia or syndrome alzheimer or dementia presenile or alzheimer disease assessment scale or alzheimer's disease or alzheimer's-disease or alzheimer-disease).kw.
3. exp Dementia/ or dementia.sh.
4. exp dementia assessment/ or exp presenile dementia/ or exp senile dementia/ or (dementia assessment or presenile dementia or senile dementia).sh.
5. (amentia* or dement* senile paranoid or dementia presenile or mental deterioration).tw. or (amentia* or dement* senile paranoid or dementia presenile or mental deterioration).kw.
6. 1 or 2 or 3 or 4 or 5

2) Observational/Prognosis/Predictor/Comorbidity

7. "Observational studies".ti. or epidemiologic studies/ or exp case-control studies/ or cross-sectional studies/
8. ((case adj3 control) or (cohort adj5 (study or studies or analy$)) or (follow-up adj5 (study or studies)) or (longitudinal or retrospective or prospective or (cross adj5 sectional)) or (observational adj5 (study or studies))).af.
9. 7 or 8
10. 6 and 9
11. exp Probability/ or probab*.sh.
12. exp Prediction/ or predict*.sh.
13. exp risk factor/ or risk factor*.sh.
14. exp Prognosis/ or prognos*.sh.
15. exp "prediction and forecasting"/ or exp forecasting/ or ((prediction and forecasting) or forecasting).sh.
16. (decision support techniques or prediction or predictive validity or predictor variable).tw. or (decision support techniques or prediction or predictive validity or predictor variable).kw.
17. ((risk adj prediction) or (predictor adj variabl??) or (increas* adj risk)).mp. or ((risk adj assessement?) or (predict* adj risk?) or (risk adj factor?) or (validat* or predict* or rule*)).tw. or

(((risk adj prediction) or (predictor adj variabl??) or (increas* adj risk)).mp. or ((risk adj assessement?) or (predict* adj risk?) or (risk adj factor?) or (validat* or predict* or rule*)).kw.)
18. ((predict* and (outcome* or risk* or model*)) or ((history or variable* or criteria or scor* or characteristic* or finding* or factor*) and (predict* or model* or decision* or identi* or prognos*)) or (decision* and (model* or clinical* or (logistic adj3 models)))).tw. or ((predict* and (outcome* or risk* or model*)) or ((history or variable* or criteria or scor* or characteristic* or finding* or factor*) and (predict* or model* or decision* or identi* or prognos*)) or (decision* and (model* or clinical* or (logistic adj3 models)))).kw.
19. 11 or 12 or 13 or 14 or 15 or 16 or 17 or 18
20. exp Comorbidity/ or comorbidit*.sh.
21. exp comorbidity assessment/ or comorbidity assessment.sh.
22. (multimorbidit* or comorbidit*).tw. or (multimorbidit* or comorbidit*).kw.
23. (co morbidit* or co-morbidit* or coomorbidit*).tw. or (co morbidit* or co-morbidit* or coomorbidit*).kw.
24. (chronic diseases or cooccurring diseases or co-occurring diseases or co occurring diseases or clusters of diseases or disease burden or physical health or medical health or charlson or cumulative illness scale geriatrics or polymorbidity or disease count).tw. or (chronic diseases or cooccurring diseases or co-occurring diseases or co occurring diseases or clusters of diseases or disease burden or physical health or medical health or charlson or cumulative illness scale geriatrics or polymorbidity or disease count).kw.
25. 20 or 21 or 22 or 23 or 24
26. 19 and 25

3) Multidimensional progression

27. exp Cognition/ or cognit*.sh.
28. exp mild cognitive impairment/ or exp cognitive defect/ or (mild cognitive impairment or cognitive defect).sh.
29. exp cognition assessment/ or cognitive assessment.sh.
30. cognit*.tw. or cognit*.kw.
31. (consciousness disorders or overinclusion or cognitive performance or confusion or intellectual disability or perceptual disorders or mental competency or perception or thinking or aptitude or mild cognitive impairment or cognitive defect or cognitive generalization or cognitive complexity or cognitive contiguity or cognitive dissonance or cognitive appraisal or cognitive maps or wayfinding or spatial imagery or direction perception or thought content or cognitive functioning or executive functioning or intellectual functioning or mathematical ability or reading ability or verbal ability or cognitive deficits or cognitive dysfunction or executive dysfunction or thought disturbances or human information process or cognitive science or information processing model or metacognition or intelligence or intelligence measures or intelligence quotient or cognitive disorders).tw. or (consciousness disorders or overinclusion or cognitive performance or confusion or intellectual disability or perceptual disorders or mental competency or perception or thinking or aptitude or mild cognitive impairment or cognitive defect or cognitive generalization or cognitive complexity or cognitive contiguity or cognitive dissonance or cognitive appraisal or cognitive maps or wayfinding or spatial imagery or direction perception or thought content or cognitive functioning or executive functioning or intellectual functioning or mathematical ability or reading ability or verbal ability or cognitive deficits or cognitive dysfunction or executive dysfunction or thought disturbances or human information process or cognitive science or information processing model or metacognition or intelligence or intelligence measures or intelligence quotient or cognitive disorders).kw.
32. exp memory/ or memory.sh.
33. exp short term memory/ or exp sensory memory/ or exp spatial memory test/ or exp explicit memory/ or exp memory disorder/ or exp memory bias/ or exp associative memory/ or exp false

memory/ or exp episodic memory/ or exp auditory memory/ or exp autobiographical memory/ or exp retrospective memory/ or exp verbal memory/ or exp working memory/ or exp implicit memory/ or exp prospective memory/ or exp memory assessment/ or exp memory consolidation/ or exp long term memory/ or exp olfactory memory/ or exp spatial memory/ or exp visual memory/ or exp tactile memory/ or exp reference memory/ or exp semantic memory/ or (short term memory or sensory memory or spatial memory test or explicit memory or memory disorder or memory bias or associative memory or false memory or episodic memory or auditory memory or autobiographical memory or retrospective memory or verbal memory or working memory or implicit memory or prospective memory or memory assessment or memory consolidation or long term memory or olfactory memory or spatial memory or visual memory or tactile memory or reference memory or semantic memory).sh.
34. (spatial memory disorders or memory disorder or memory losses or memory disorders age related or retention disorder cognitive or memory disorder semantic or age-related memory disorder or memory deficits or spatial memory disorder or cognitive retention disorder or immediate memories or working memory or memory shortterm or recall immediate or amnesia or anterograde amnesia or global amnesia or retrograde amnesia or memory decay or memory trace or visuospatial memory or associative memory or auditory memory or false memory or olfactory memory or recognition or reference memory or repetition priming or retrospective memory or sensory memory or tactile memory or memory bias or word list recall or word recognition or working memory).tw. or (spatial memory disorders or memory disorder or memory losses or memory disorders age related or retention disorder cognitive or memory disorder semantic or age-related memory disorder or memory deficits or spatial memory disorder or cognitive retention disorder or immediate memories or working memory or memory shortterm or recall immediate or amnesia or anterograde amnesia or global amnesia or retrograde amnesia or memory decay or memory trace or visuospatial memory or associative memory or auditory memory or false memory or olfactory memory or recognition or reference memory or repetition priming or retrospective memory or sensory memory or tactile memory or memory bias or word list recall or word recognition or working memory).kw.
35. exp language/ or language.sh.
36. exp speech/ or speech.sh.
37. exp speech language pathologist/ or exp language ability/ or exp language disability/ or exp "speech and language"/ or exp "speech and language assessment"/ or ((((speech language pathologist or language ability or language disability or speech) and language) or speech) and language assessment).sh.
38. exp speech disorder/ or exp speech analysis/ or exp speech perception/ or (speech disorder or speech analysis or speech perception).sh.
39. (language disorders or language disorder acquired or pathology speech or pathology language or verbal fluency or linguistic or oral communication or communication disorders).tw. or (language disorders or language disorder acquired or pathology speech or pathology language or verbal fluency or linguistic or oral communication or communication disorders).kw.
40. exp Verbal Behavior/ or verbal behavior.sh.
41. exp Executive Function/ or executive function*.sh.
42. exp Attention/ or attention.sh.
43. exp Problem Solving/ or problem solving.sh.
44. exp Decision Making/ or decision making.sh.
45. exp Orientation/ or orientation.sh.
46. exp Reading/ or reading.sh.
47. exp Judgment/ or judgment.sh.
48. exp mental concentration/ or mental concentration.sh.
49. exp mental capacity/ or mental capacity.sh.
50. exp aptitude/ or aptitude.sh.

51. (executive control or concentration or shared decision making or mental speed or verbal reasoning or abstraction).tw. or (executive control or concentration or shared decision making or mental speed or verbal reasoning or abstraction).kw.
52. 27 or 28 or 29 or 30 or 31 or 32 or 33 or 34 or 35 or 36 or 37 or 38 or 39 or 40 or 41 or 42 or 43 or 44 or 45 or 46 or 47 or 48 or 49 or 50 or 51

53. exp daily life activity/ or daily life activity.sh.
54. exp physical mobility/ or exp physical capacity/ or exp physical performance/ or exp physical activity/ or exp physical disability/ or (physical mobility or physical capacity or physical performance or physical activity or physical disability).sh.
55. exp motor coordination/ or exp motor control/ or exp motor dysfunction/ or exp motor performance/ or exp motor activity/ or (motor coordination or motor control or motor dysfunction or motor performance or motor activity).sh.
56. exp Locomotion/ or locomot*.sh.
57. exp walking/ or exp walking speed/ or exp walking difficulty/ or (walking or walking speed or walking difficulty).sh.
58. exp gait/ or exp unsteady gait/ or exp gait disorder/ or (gait or unsteady gait or gait disorder).sh.
59. exp endurance/ or endurance.sh.
60. exp balance disorder/ or exp balance impairment/ or (balance disorder or balance impairment).sh.
61. exp body equilibrium/ or body equilibrium.sh.
62. exp falling/ or falling.sh.
63. exp grip strength/ or exp pinch strength/ or exp hand strength/ or exp muscle strength/ or exp grip strength test/ or exp strength/ or (grip strength or pinch strength or hand strength or muscle strength or grip strength test or strength).sh.
64. exp Self Care/ or self care.sh.
65. exp personal autonomy/ or exp personal hygiene/ or (personal autonomy or personal hygiene).sh.
66. exp work disability/ or exp work capacity/ or (work disability or work capacity).sh.
67. (physical function or ambulation or limitation of activity chronic or self management or self-care or self-management or musculoskeletal equilibrium or postural equilibrium or grasps or grips or pinch strength or (physical function or ambulation or limitation of activity chronic or self management or self-care or self-management or musculoskeletal equilibrium or postural equilibrium or grasps or grips or pinch strength) or (physical function or ambulation or limitation of activity chronic or self management or self-care or self-management or musculoskeletal equilibrium or postural equilibrium or grasps or grips or pinch strength)).tw. or (physical function or ambulation or limitation of activity chronic or self management or self-care or self-management or musculoskeletal equilibrium or postural equilibrium or grasps or grips or pinch strength).kw.
68. 53 or 54 or 55 or 56 or 57 or 58 or 59 or 60 or 61 or 62 or 63 or 64 or 65 or 66 or 67

69. exp mental instability/ or mental instability.sh.
70. exp psychiatry/ or psychiatr*.sh.
71. exp gerontopsychiatry/ or gerontopsychiatr*.sh.
72. exp neuropsychiatry/ or neuropsychiat*.sh.
73. exp behavior control/ or exp illness behavior/ or exp behavior disorder assessment/ or exp behavior assessment/ or exp behavior change/ or exp behavior modification/ or exp behavior disorder/ or (behavior control or illness behavior or behavior disorder assessment or behavior assessment or behavior change or behavior modification or behavior disorder).sh.

74. exp anxiety assessment/ or exp Depression Anxiety Stress Scale/ or exp anxiety/ or exp anxiety disorder/ or exp generalized anxiety disorder/ or exp "mixed anxiety and depression"/ or ((anxiety assessment or Depression Anxiety Stress Scale or anxiety or anxiety disorder or generalized anxiety disorder or mixed anxiety) and depression).sh.
75. exp confusion/ or confusion.sh.
76. exp delirium/ or delirium.sh.
77. exp emotional disorder/ or emotional disorder.sh.
78. exp mood disorder assessment/ or exp mood change/ or exp mood disorder/ or (mood disorder assessment or mood change or mood disorder).sh.
79. exp organic psychosyndrome/ or organic psychosyndrome.sh.
80. exp aggression/ or exp hostility/ or (aggression or hostility).sh.
81. exp anger/ or anger.sh.
82. exp Apathy/ or apathy.sh.
83. exp Depression/ or depression.sh.
84. exp dysphoria/ or dysphoria.sh.
85. exp delusion/ or delusion.sh.
86. exp auditory hallucination/ or exp hallucination/ or exp visual hallucination/ or (auditory hallucination or hallucination or visual hallucination).sh.
87. exp agitation/ or exp agitation assessment/ or (agitation or agitation assessment).sh.
88. exp Euphoria/ or euphoria.sh.
89. exp Irritability/ or irritability.sh.
90. (mental patient or mental disorder or neuropsychological assessment or psychiatric evaluation or psychiatric disorders or indifference or disinhibition or lability or aberrant motor behavior or hallucinat* or agitat* or anxiety elation or apath* or neuropsychiat*).tw. or (mental patient or mental disorder or neuropsychological assessment or psychiatric evaluation or psychiatric disorders or indifference or disinhibition or lability or aberrant motor behavior or hallucinat* or agitat* or anxiety elation or apath* or neuropsychiat*).kw.
91. 69 or 70 or 71 or 72 or 73 or 74 or 75 or 76 or 77 or 78 or 79 or 80 or 81 or 82 or 83 or 84 or 85 or 86 or 87 or 88 or 89 or 90

92. exp Disease Course/ or disease course.sh.
93. exp disease control/ or disease control.sh.
94. exp outcome assessment/ or exp adverse outcome/ or (outcome assessment or adverse outcome).sh.
95. exp chronic disease/ or exp chronic patient/ or (chronic disease or chronic patient).sh.
96. exp chronicity/ or chronicity.sh.
97. exp survival rate/ or exp survival/ or exp survival prediction/ or (survival rate or survival or survival prediction).sh.
98. exp onset age/ or onset age.sh.
99. exp Terminal Care/ or terminal care.sh.
100. exp disease duration/ or disease duration.sh.
101. exp general condition deterioration/ or exp deterioration/ or exp mental deterioration/ or (general condition deterioration or deterioration or mental deterioration).sh.
102. exp disease exacerbation/ or disease exacerbation.sh.
103. exp general condition improvement/ or general condition improvement.sh.
104. exp illness trajectory/ or illness trajectory.sh.
105. (disease control or disease progression or remission or progress* or impairment or decline or failure or decrease or worsening or deterioration or degeneration).tw. or (disease control or disease

progression or remission or progress* or impairment or decline or failure or decrease or worsening or deterioration or degeneration).kw.
106. 92 or 93 or 94 or 95 or 96 or 97 or 98 or 99 or 100 or 101 or 102 or 103 or 104 or 105

Final combinations:

107. 10 and 25 and 106
108. 52 and 107
109. 68 and 107
110. 91 and 107

11. Appendix 3
Search Cochrane

1) Alzheimer's Disease

#1 MeSH descriptor: [Alzheimer Disease] explode all trees

#2 alzheimer sclerosis or alzheimer disease late onset or alzheimer type dementia or dementia primary senile degenerative or presenile alzheimer dementia or alzheimer syndrome or dementia alzheimer-type or senile dementia or dementia alzheimer or dementia alzheimer type or presenile dementia or dementia senile or late onset alzheimer disease or alzheimer's disease or primary senile degenerative dementia or syndrome alzheimer or dementia presenile or alzheimer disease assessment scale or alzheimer's disease or alzheimer's-disease or alzheimer-disease

#3 MeSH descriptor: [Dementia] explode all trees

#4 amentia* or dement* senile paranoid or dementia presenile or mental deterioration

#5 #1 or #2 or #3 or #4

2) Observational/Prognosis/Predictor/Comorbidity

#6 MeSH descriptor: [Patients] explode all trees

#7 Observational studies or epidemiologic studies or exp case-control studies or cross-sectional studies or (case adj3 control) or (cohort adj5 (study or studies or analy$)) or (follow-up adj5 (study or studies)) or (longitudinal or retrospective or prospective or (cross adj5 sectional)) or (observational adj5 (study or studies))

#8 #6 or #7

#9 #5 and #8

#10 MeSH descriptor: [Prognosis] explode all trees

#11 MeSH descriptor: [Probability] explode all trees

#12 MeSH descriptor: [Forecasting] explode all trees

#13 MeSH descriptor: [Risk Factors] explode all trees

#14 MeSH descriptor: [Decision Support Techniques] explode all trees

#15 prediction or predictive validity or predictor variable

#16 (risk adj prediction) or (predictor adj variabl??) or (increas* adj risk) or (risk adj assessement?) or (predict* adj risk?) or (risk adj factor?) or (validat* or predict* or rule*)

#17 ((predict* and (outcome* or risk* or model*)) or ((history or variable* or criteria or scor* or characteristic* or finding* or factor*) and (predict* or model* or decision* or identi* or prognos*)) or (decision* and (model* or clinical* or (logistic adj3 models))))

#18 #10 or #11 or #12 or #13 or #14 or #15 or #16 or #17

#19 MeSH descriptor: [Comorbidity] explode all trees

#20 MeSH descriptor: [Chronic Disease] explode all trees

#21 (multimorbidit* or comorbidit*)

#22 (co morbidit* or co-morbidit* or coomorbidit*)

#23 (cooccurring diseases or co-occurring diseases or co occurring diseases or clusters of diseases or comorbidity assessment or disease burden or physical health or medical health or charlson or cumulative illness scale geriatrics or polymorbidity or disease count)

#24 #19 or #20 or #21 or #22 or #23

#25 #18 and #24

3) **Multidimensional progression**

#26 MeSH descriptor: [Cognition] explode all trees

#27 MeSH descriptor: [Cognition Disorders] explode all trees

#28 MeSH descriptor: [Confusion] explode all trees

#29 MeSH descriptor: [Consciousness Disorders] explode all trees

#30 MeSH descriptor: [Intellectual Disability] explode all trees
#31 MeSH descriptor: [Perception] explode all trees
#32 MeSH descriptor: [Perceptual Disorders] explode all trees
#33 MeSH descriptor: [Mental Competency] explode all trees
#34 overinclusion or cognitive performance or thinking or aptitude or mild cognitive impairment or cognitive defect or cognitive generalization or cognitive complexity or cognitive contiguity or cognitive dissonance or cognitive appraisal or cognitive maps or wayfinding or spatial imagery or direction perception or thought content or cognitive functioning or executive functioning or intellectual functioning or mathematical ability or reading ability or verbal ability or cognitive deficits or cognitive dysfunction or executive dysfunction or thought disturbances or human information process or cognitive science or information processing model or metacognition or intelligence or intelligence measures or intelligence quotient or cognitive disorders or mild cognitive impairment or cognitive defect or cognition assessment
#35 cognit*
#36 MeSH descriptor: [Aptitude] explode all trees
#37 MeSH descriptor: [Thinking] explode all trees
#38 MeSH descriptor: [Attention] explode all trees
#39 MeSH descriptor: [Orientation] explode all trees
#40 MeSH descriptor: [Memory] explode all trees
#41 MeSH descriptor: [Memory Disorders] explode all trees
#42 short term memory or sensory memory or spatial memory test or explicit memory or memory bias or associative memory or false memory or episodic memory or auditory memory or autobiographical memory or retrospective memory or verbal memory or working memory or implicit memory or prospective memory or memory assessment or memory consolidation or long term memory or olfactory memory or spatial memory or visual memory or tactile memory or reference memory or semantic memory or spatial memory disorders or memory losses or memory disorders age related or retention disorder cognitive or memory disorder semantic or age-related memory disorder or memory deficits or spatial memory disorder or cognitive retention disorder or immediate memories or working memory or memory shortterm or recall immediate or amnesia or anterograde amnesia or global amnesia or retrograde amnesia or memory decay or memory trace or visuospatial memory or associative memory or auditory memory or false memory or olfactory memory or recognition or reference memory or repetition priming or retrospective memory or sensory memory or memory bias or word list recall or word recognition or working memory
#43 MeSH descriptor: [Language] explode all trees
#44 MeSH descriptor: [Language Disorders] explode all trees
#45 MeSH descriptor: [Speech] explode all trees
#46 MeSH descriptor: [Speech Disorders] explode all trees
#47 MeSH descriptor: [Communication Disorders] explode all trees
#48 speech language pathologist or language ability or language disability or "speech and language" or "speech and language assessment" or speech analysis or speech perception or language disorder acquired or pathology speech or pathology language or verbal fluency or linguistic or oral communication
#49 MeSH descriptor: [Verbal Behavior] explode all trees
#50 MeSH descriptor: [Executive Function] explode all trees
#51 MeSH descriptor: [Decision Making] explode all trees
#52 MeSH descriptor: [Problem Solving] explode all trees
#53 MeSH descriptor: [Reading] explode all trees
#54 MeSH descriptor: [Judgment] explode all trees
#55 executive control or concentration or shared decision making or mental speed or verbal reasoning or abstraction

#56 #26 or #27 or #28 or #29 or #30 or #31 or #32 or #33 or #34 or #35 or #36 or #37 or #38 or #39 or #40 or #41 or #42 or #43 or #44 or #45 or #46 or #47 or #48 or #49 or #50 or #51 or #52 or #53 or #54 or #55

#57 MeSH descriptor: [Activities of Daily Living] explode all trees
#58 MeSH descriptor: [Exercise] explode all trees
#59 MeSH descriptor: [Motor Activity] explode all trees
#60 MeSH descriptor: [Physical Endurance] explode all trees
#61 MeSH descriptor: [Motor Skills Disorders] explode all trees
#62 MeSH descriptor: [Locomotion] explode all trees
#63 MeSH descriptor: [Gait Disorders, Neurologic] explode all trees
#64 MeSH descriptor: [Walking] explode all trees
#65 MeSH descriptor: [Psychomotor Disorders] explode all trees
#66 MeSH descriptor: [Movement Disorders] explode all trees
#67 MeSH descriptor: [Accidental Falls] explode all trees
#68 MeSH descriptor: [Postural Balance] explode all trees
#69 MeSH descriptor: [Muscle Strength] explode all trees
#70 MeSH descriptor: [Pinch Strength] explode all trees
#71 MeSH descriptor: [Hand Strength] explode all trees
#72 MeSH descriptor: [Compressive Strength] explode all trees
#73 MeSH descriptor: [Work Capacity Evaluation] explode all trees
#74 MeSH descriptor: [Self Care] explode all trees
#75 MeSH descriptor: [Personal Autonomy] explode all trees
#76 living assessment or lifting effort or weight bearing or weight lifting or writing or physical inactivity or physical performance or physical capacity or exercise tolerance or motor performance or object manipulation or self help or self concept or self control or personal hygiene or personal needs or psychomotor activity or driving ability or gesture or handedness or handwriting or self stimulation or skill or psychomotor disorder or unsteady gait or motor dysfunction or motor coordination or motor dysfunction assessment or motor control or body equilibrium or fall risk or fall risk assessment or falling or ability level or activity level or assisted living or daily activities or habilitation or independent living programs or physical mobility or self care skills or physical function or ambulation or limitation of activity chronic or self management or self-care or self-management or musculoskeletal equilibrium or equilibrium postural or grasp*
#77 #57 or #58 or #59 or #60 or #61 or #62 or #63 or #64 or #65 or #66 or #67 or #68 or #69 or #70 or #71 or #72 or #73 or #74 or #75 or #76

#78 MeSH descriptor: [Neuropsychiatry] explode all trees
#79 MeSH descriptor: [Neurobehavioral Manifestations] explode all trees
#80 MeSH descriptor: [Mental Disorders] explode all trees
#81 MeSH descriptor: [Behavior Control] explode all trees
#82 MeSH descriptor: [Behavioral Symptoms] explode all trees
#83 MeSH descriptor: [Affective Symptoms] explode all trees
#84 MeSH descriptor: [Mood Disorders] explode all trees
#85 MeSH descriptor: [Psychiatry] explode all trees
#86 MeSH descriptor: [Geriatric Psychiatry] explode all trees
#87 MeSH descriptor: [Neuropsychiatry] explode all trees
#88 MeSH descriptor: [Anxiety Disorders] explode all trees
#89 MeSH descriptor: [Confusion] explode all trees
#90 MeSH descriptor: [Delirium] explode all trees
#91 MeSH descriptor: [Aggression] explode all trees

#92 MeSH descriptor: [Irritable Mood] explode all trees
#93 MeSH descriptor: [Hostility] explode all trees
#94 MeSH descriptor: [Anger] explode all trees
#95 MeSH descriptor: [Apathy] explode all trees
#96 MeSH descriptor: [Depression] explode all trees
#97 MeSH descriptor: [Delusions] explode all trees
#98 MeSH descriptor: [Hallucinations] explode all trees
#99 MeSH descriptor: [Psychomotor Agitation] explode all trees
#100 mental patient or mental disorder or neuropsychological assessment or psychiatric evaluation or psychiatric disorders or indifference or disinhibition or lability or aberrant motor behavior or hallucinat* or agitat* or dysphoria or anxiety elation or apath* or neuropsychiat*
#101 #78 or #79 or #80 or #81 or #82 or #83 or #84 or #85 or #86 or #87 or #88 or #89 or #90 or #91 or #92 or #93 or #94 or #95 or #96 or #97 or #98 or #99 or #100

#102 MeSH descriptor: [Disease Progression] explode all trees
#103 MeSH descriptor: [Outcome and Process Assessment (Health Care)] explode all trees
#104 MeSH descriptor: [Patient Outcome Assessment] explode all trees
#105 MeSH descriptor: [Survival Analysis] explode all trees
#106 MeSH descriptor: [Age of Onset] explode all trees
#107 MeSH descriptor: [Chronic Disease] explode all trees
#108 disease course or disease control or adverse outcome or chronic patient or chronicity or survival rate or survival or survival prediction or terminal care or disease duration or general condition deterioration or deterioration or mental deterioration or disease exacerbation or general condition improvement or illness trajectory or remission or progress* or impairment or decline or failure or decrease or worsening or deterioration or degeneration
#109 #102 or #103 or #104 or #105 or #106 or #107 or #108

Final combinations:

#110 #9 and #25 and #109
#111 #110 and #56
#112 #110 and #77
#113 #110 and #101

12. Appendix 4

PROGNOSIS WORKSHEET

Citation:

Are the results of this prognosis study valid?

Citation:

Was a defined, representative sample of patients assembled at a common (usually early) point in the course of their disease?	
Was patient follow-up sufficiently long and complete?	
Were objective outcome criteria applied in a "blind" fashion?	
If subgroups with different prognoses are identified, was there adjustment for important prognostic factors?	
Was there validation in an independent group ("test set") of patients?	

Are the valid results of this prognosis study important?

Citation:

How likely are the outcomes over time?	
How precise are the prognostic estimates?	

Can you apply this valid, important evidence about prognosis in caring for your patient?

Were the study patients similar to your own?	
Will this evidence make a clinically important impact on your conclusions about what to offer or tell your patient?	

Additional notes:

13. References

1. Chertkow, H., et al., *Definitions of dementia and predementia states in Alzheimer's disease and vascular cognitive impairment: consensus from the Canadian conference on diagnosis of dementia.* Alzheimers Res Ther, 2013. **5**(Suppl 1): p. S2.
2. International, W.H.O.A.s.D., *Dementia: A public health priority.* 2012.
3. Savva, G.M., et al., *Age, neuropathology, and dementia.* N Engl J Med, 2009. **360**(22): p. 2302-9.
4. Sona, A., K.A. Ellis, and D. Ames, *Rapid cognitive decline in Alzheimer's disease: A literature review.* International Review of Psychiatry, 2013. **.25**(6): p. pp.
5. Oosterveld, S.M., et al., *The influence of co-morbidity and frailty on the clinical manifestation of patients with Alzheimer's disease.* J Alzheimers Dis, 2014. **42**(2): p. 501-9.
6. Rosenstein, L.D., *Differential diagnosis of the major progressive dementias and depression in middle and late adulthood: a summary of the literature of the early 1990s.* Neuropsychol Rev, 1998. **8**(3): p. 109-67.
7. Aguero-Torres, H., L. Fratiglioni, and B. Winblad, *Natural history of Alzheimer's disease and other dementias: review of the literature in the light of the findings from the Kungsholmen Project.* Int J Geriatr Psychiatry, 1998. **13**(11): p. 755-66.
8. Omran, A.R., *The epidemiologic transition: a theory of the epidemiology of population change. 1971.* Milbank Q, 2005. **83**(4): p. 731-57.
9. Salive, M.E., *Multimorbidity in older adults.* Epidemiol Rev, 2013. **35**: p. 75-83.
10. Doraiswamy, P.M., et al., *Prevalence and impact of medical comorbidity in Alzheimer's disease.* J Gerontol A Biol Sci Med Sci, 2002. **57**(3): p. M173-7.
11. Middleton, L.E. and K. Yaffe, *Promising strategies for the prevention of dementia.* Arch Neurol, 2009. **66**(10): p. 1210-5.
12. Melis, R.J., et al., *The influence of multimorbidity on clinical progression of dementia in a population-based cohort.* PLoS One, 2013. **8**(12): p. e84014.
13. Davidson, J.E., et al., *An exploration of cognitive subgroups in Alzheimer's disease.* J Int Neuropsychol Soc, 2010. **16**(2): p. 233-43.
14. Backman, L., et al., *Rate of cognitive decline in preclinical Alzheimer's disease: the role of comorbidity.* J Gerontol B Psychol Sci Soc Sci, 2003. **58**(4): p. P228-36.
15. Leoutsakos, J.M., et al., *Effects of general medical health on Alzheimer's progression: the Cache County Dementia Progression Study.* Int Psychogeriatr, 2012. **24**(10): p. 1561-70.
16. MacDonald, S.W., et al., *Trajectories of cognitive decline following dementia onset: what accounts for variation in progression?* Dement Geriatr Cogn Disord, 2011. **31**(3): p. 202-9.
17. Solomon, A., et al., *Comorbidity and the rate of cognitive decline in patients with Alzheimer dementia.* Int J Geriatr Psychiatry, 2011. **26**(12): p. 1244-51.
18. Jakovljevic, M. and L. Ostojic, *Comorbidity and multimorbidity in medicine today: challenges and opportunities for bringing separated branches of medicine closer to each other.* Psychiatr Danub, 2013. **25 Suppl 1**: p. 18-28.
19. Brandimonte, M.A., N. Bruno, and S. Collina, *Chapter: Cognition*, in *Psychological concepts: An international historical perspective*. 2006, Psychology Press/Taylor & Francis (UK); England: Hove, England. p. 11-26.
20. Gold, D.A., *An examination of instrumental activities of daily living assessment in older adults and mild cognitive impairment.* J Clin Exp Neuropsychol, 2012. **34**(1): p. 11-34.
21. Miyoshi, K. and Y. Morimura, *Chapter: Clinical manifestations of neuropsychiatric disorders*, in *Neuropsychiatric disorders*. 2010, Springer Science + Business Media; US: New York, NY. p. 3-15.
